# Regulation of the Dimerization and Activity of SARS-CoV-2 Main Protease through Reversible Glutathionylation of Cysteine 300

**DOI:** 10.1128/mBio.02094-21

**Published:** 2021-08-17

**Authors:** David A. Davis, Haydar Bulut, Prabha Shrestha, Amulya Yaparla, Hannah K. Jaeger, Shin-ichiro Hattori, Paul T. Wingfield, John J. Mieyal, Hiroaki Mitsuya, Robert Yarchoan

**Affiliations:** a HIV and AIDS Malignancy Branch, Center for Cancer Research, National Cancer Institute, Bethesda, Maryland, USA; b National Institute of Arthritis and Musculoskeletal and Skin Diseases, National Institutes of Health, Bethesda, Maryland, USA; c Department of Refractory Viral Infections, National Center for Global Health and Medicine Research Institute, Tokyo, Japan; d Department of Pharmacology, Case Western Reserve University School of Medicine, Cleveland, Ohio, USA; University of North Carolina, Chapel Hill

**Keywords:** COVID-19, SARS-CoV-2, dimerization, drug targets, glutaredoxin, glutathionylation, main protease, oxidative stress, thioltransferase

## Abstract

Severe acute respiratory syndrome coronavirus 2 (SARS-CoV-2), the causative agent for coronavirus disease 2019 (COVID-19), encodes two proteases required for replication. The main protease (M^pro^), encoded as part of two polyproteins, pp1a and pp1ab, is responsible for 11 different cleavages of these viral polyproteins to produce mature proteins required for viral replication. M^pro^ is therefore an attractive target for therapeutic interventions. Certain proteins in cells under oxidative stress undergo modification of reactive cysteines. We show M^pro^ is susceptible to glutathionylation, leading to inhibition of dimerization and activity. Activity of glutathionylated M^pro^ could be restored with reducing agents or glutaredoxin. Analytical studies demonstrated that glutathionylated M^pro^ primarily exists as a monomer and that modification of a single cysteine with glutathione is sufficient to block dimerization and inhibit its activity. Gel filtration studies as well as analytical ultracentrifugation confirmed that glutathionylated M^pro^ exists as a monomer. Tryptic and chymotryptic digestions of M^pro^ as well as experiments using a C300S M^pro^ mutant revealed that Cys300, which is located at the dimer interface, is a primary target of glutathionylation. Moreover, Cys300 is required for inhibition of activity upon M^pro^ glutathionylation. These findings indicate that M^pro^ dimerization and activity can be regulated through reversible glutathionylation of a non-active site cysteine, Cys300, which itself is not required for M^pro^ activity, and provides a novel target for the development of agents to block M^pro^ dimerization and activity. This feature of M^pro^ may have relevance to the pathophysiology of SARS-CoV-2 and related bat coronaviruses.

## INTRODUCTION

The main protease (M^pro^) of severe acute respiratory syndrome coronavirus 2 (SARS-CoV-2) is encoded as part of two large polyproteins, pp1a and pp1ab, and is responsible for 11 different cleavages during initial stages of viral replication. Thus, M^pro^ is essential and has been identified as a promising target for the development of therapeutics for treatment of coronavirus disease 2019 (COVID-19) (1, 2). M^pro^ is known as a 3C-like protease (3CL) due to its similarity to picornavirus 3C protease in its cleavage site specificity (3). Through prior extensive studies on M^pro^ from SARS-CoV-1, whose sequence is 96% identical to SARS-CoV-2 M^pro^, a wealth of information is available that can be applied to studies now ongoing with SARS-CoV-2 M^pro^ (for a review, see reference 4). M^pro^ of SARS-CoV-1 and SARS-CoV-2 consists of three major domains, I, II, and III. Unlike other 3C-like proteases, studies of M^pro^ from SARS-CoV-1 and SARS-CoV-2 have revealed that they are only active as homodimers even though each individual monomeric subunit contains its own active site (5, 6). Studies of SARS-CoV-1 to uncover why dimerization is required for activity have revealed that, in the monomeric state, the active site pocket collapses and is not available for substrate binding and processing (7). In these studies, it was also revealed that the extra domain (III) plays a key role in dimerization and activation of M^pro^ and that arginine 298 in this domain is essential to allow proper dimerization and M^pro^ activity (7).

Like M^pro^, the proteases of HIV and other retroviruses are also active as homodimers, and we previously demonstrated that the retroviral proteases studied from HIV-1, HIV-2, and human T-cell leukemia virus 1 (HTLV-1) could be regulated through reversible oxidation of a cysteine or methionine residue in the dimerization domain (8–11). Modification of these residues leads to inhibition of dimerization and therefore activity (8, 12). Importantly, glutathionylation of cysteine 95 (the formation of a disulfide bond between glutathione and cysteine 95 residue) of HIV-1 protease was reversible using the cellular enzyme glutaredoxin (Grx) (13). In a similar fashion, HIV-2 protease was inhibited by oxidation of methionine 95, and this was reversible with methionine sulfoxide reductase (14). In fact, most retroviral proteases examined have one or more cysteine and/or methionine residues at the predicted dimer interface region, and modification of these residues would be predicted to similarly inhibit dimerization and activity (8).

Multiple reports describe reactive oxygen/nitrogen species (ROS/RNS) production induced by viral infections, including influenza A, hepatitis C, Sendai, respiratory syncytial viruses (15–18), and SARS-CoV-2 (for a review, see reference 19 and references therein). Certain cysteine residues on proteins are susceptible to modification by ROS/RNS, which can cause the formation of glutathionylated mixed disulfides of numerous cellular proteins, including hemoglobin, nuclear factor 1, PTP1B, actin, Ras, IκB kinase, procaspase 3, and IRF-3 (20). Importantly, protein-*S*-glutathionylation is specifically reversed by glutaredoxin (Grx). M^pro^ contains an active site cysteine (Cys145). In addition, SARS-CoV-1 and SARS-CoV-2 contain 11 other cysteine residues, and all these residues are present in their reduced form in the crystal structures of M^pro^. Given these considerations, we hypothesized that M^pro^, within an intracellular oxidative stress environment, would likely be *S*-glutathionylated, thereby affecting its function. While most cysteines are buried and may not be exceptionally susceptible to oxidation in the native structure, there are cysteine residues (cysteine 22, 85, 145, 156, and 300) that are surface exposed and potentially susceptible to oxidative modification. Here, we explored the ability to regulate M^pro^ activity through reversible oxidation. We demonstrate dimerization and activity of SARS-CoV-2 M^pro^ can be regulated through reversible glutathionylation of cysteine 300. This finding reveals a possible novel regulatory mechanism of M^pro^ and a novel target for the development of inhibitors of M^pro^ and SARS-CoV-2 replication.

## RESULTS

### Treatment of M^pro^ with oxidized glutathione inhibits protease activity, and inhibition of M^pro^ activity by glutathionylation is reversible.

**FIG 1:**
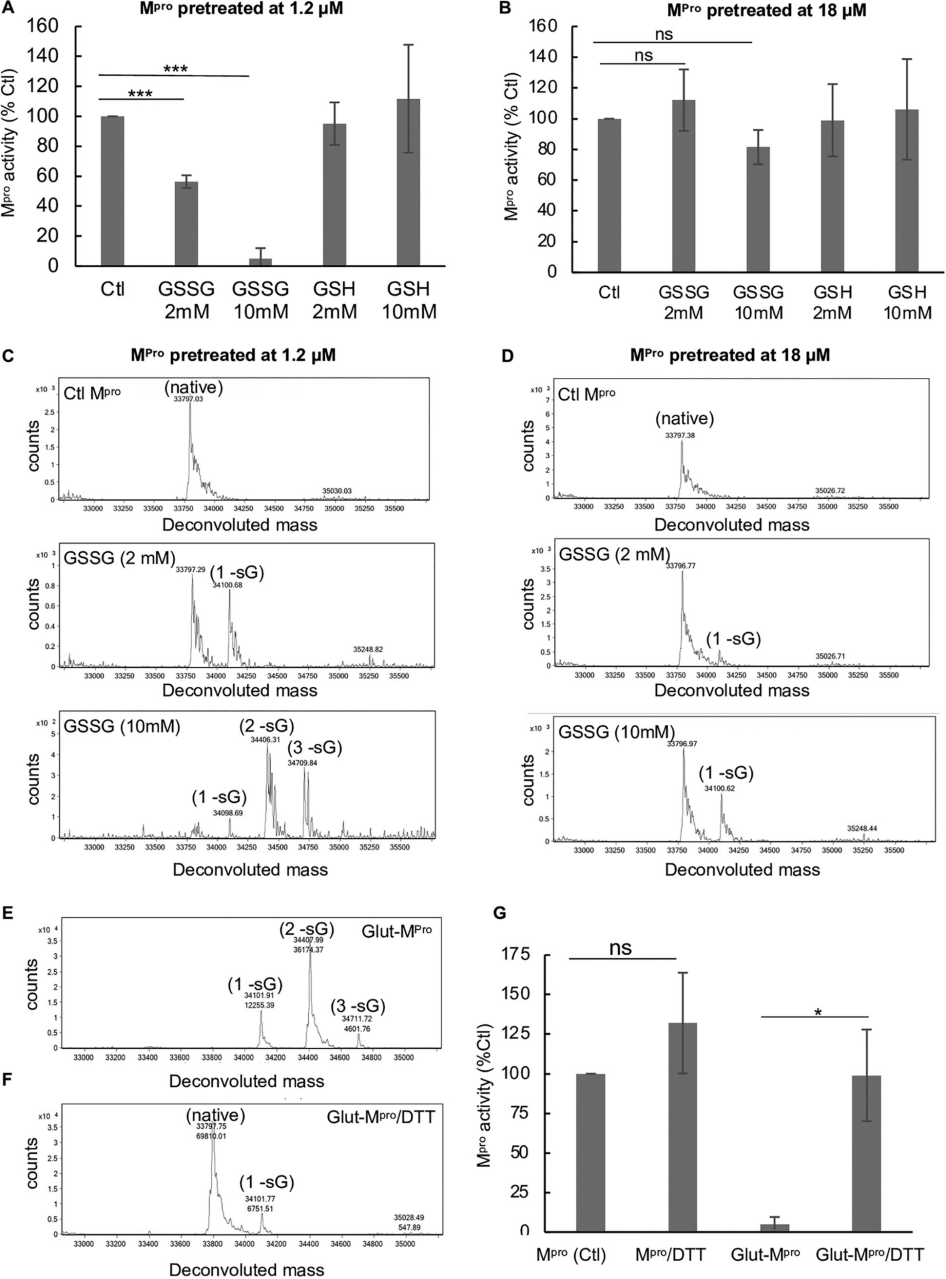
GSSG glutathionylates SARS-CoV-2 M^pro^ at low M^pro^ concentrations, resulting in inhibition of activity. (A and B) Activity of M^pro^ following a 30-min preincubation of 1.2 μM M^pro^ (A) or 18 μM M^pro^ (B) with 2 mM or 10 mM oxidized or reduced glutathione. After preincubation, M^pro^ was assayed for protease activity at an equal final enzyme concentration (1 μM). In panels A and B, the values shown are the means ± standard deviations (error bars) for three independent experiments (*n* = 3) (***, *P* value of <0.005, paired Student’s *t* test). All other comparisons to control (Ctl) activity were not found to be significant (*P* value of >0.05). M^pro^ control activity for panel A was 6.42 ± 2.5 μM/min/mg, and for panel B, it was 9.6 μM/min/mg, and the percent activity of the treatment was normalized to that of their respective control. ns, not significant. (C and D) Molecular masses found by protein deconvolution for control and GSSG-treated M^pro^ eluting from a C_18_ reverse-phase column after treatment of 1.2 μM (C) and 18 μM (D) M^pro^. The theoretical molecular mass of M^pro^ is 33,796.48, and the deconvoluted molecular masses for controls in panels C and D were 33,797.09 and 33,797.34, respectively, as determined using Agilent’s Mass Hunter software. The experimental masses are shown above each peak. The native M^pro^ as well as the increases in mass indicative of glutathionylation are indicated for the addition of one (1 -sG), two (2 -sG), and three (3 -sG) glutathione moieties in the deconvolution profiles of GSSG-treated M^pro^. The observed mass increases were 304, 609, and 913 compared to the predicted increases of 305.1, 610.2, and 915.3 for the addition of one, two, or three glutathiones, respectively. Based on the abundances, the estimated percentage of monoglutathionylation in panel C at 2 mM GSSG was 45%, and for 10 mM GSSG, there was an estimated 11% mono-, 50% di-, and 35% triglutathionylation, respectively. (D) After treatment with 2 mM GSSG, there was <5% monoglutathionylation, and with 10 mM GSSG, there was an estimated 34% monoglutathionylation. (E and F) M^pro^ (1.5 μM) was glutathionylated (Glut) at pH 7.5 with 10 mM GSSG, and then excess GSSG was removed by Amicon filtrations as described in Materials and Methods. The extent of glutathionylation was determined by RP-HPLC/MALDI-TOF MS before (E) and after (F) treatment with 10 mM DTT (30 min). Shown above each peak is the molecular mass (top number) and the abundance (bottom number). The native, monoglutathionylated (1 -sG), diglutathionylated (2 -sG), and triglutathionylated (3 -sG) M^pro^ are indicated. (G) M^pro^ activity (1 μM final enzyme) of native and glutathionylated M^pro^ (as shown in panel E) after a 30-min incubation in the presence or absence of 10 mM DTT. M^pro^ activity for control in panel G was 4.95 ± 1.2 μM/min/mg, and percent activity for the different conditions was normalized to their respective controls. The values shown are the averages ± standard deviations from three separate experiments (*n* = 3) (*, *P* value of <0.05, paired Student’s *t* test; ns, not significant).

Authentic wild-type (WT) M^pro^ prepared as described in Materials and Methods was >95% pure by sodium dodecyl sulfate (SDS)-gel electrophoresis and reverse-phase high-performance liquid chromatography (RP-HPLC) analysis. The mass was confirmed by matrix-assisted laser desorption ionization−time of flight mass spectrometry (MALDI-TOF MS) analysis (see Fig. S1A to E in the supplemental material). M^pro^ activity was measured utilizing a *para*-nitroanilide (pNA) substrate (H2N-TSAVLQ-pNA) as described previously for SARS-CoV-1 M^pro^ (21, 22). To assess the effects of oxidized glutathione (GSSG) and reduced glutathione (GSH) on M^pro^, we treated M^pro^ at concentrations of either 1.2 or 18 μM with 2 mM or 10 mM GSSG or GSH for 30 min at 37°C and then measured M^pro^ activity at 1 μM concentration for each treated sample. Previous reports have indicated that the dissociation constant (*K_d_*) of M^pro^ dimerization is about 2 μM (6) and that is consistent to what we found in our work. Thus, M^pro^ would be largely monomeric at 1.2 μM and dimeric at 18 μM. After exposure of 1.2 μM M^pro^ to 2 mM GSSG, activity was inhibited by an average of 44%, while after exposure to 10 mM GSSG, activity was inhibited by more than 90% (Fig. 1A). By contrast, GSH had little effect at these concentrations (Fig. 1A). Interestingly, when the M^pro^ concentration was increased to 18 μM, it was resistant to GSSG inhibition, with no inhibition observed with 2 mM GSSG and less than 20% inhibition with 10 mM GSSG (Fig. 1B). These results suggest that monomeric M^pro^ is more sensitive to glutathionylation than dimeric M^pro^. To confirm that M^pro^ was becoming modified with GSSG under these conditions, we acidified the samples at the end of the enzyme assays with formic acid/trifluoroacetic acid (FA/TFA) to arrest activity and glutathionylation and analyzed them by RP-HPLC/MALDI-TOF MS. The extent of glutathionylation was assessed by determining the mass of M^pro^ by protein deconvolution and by looking for the addition of approximately 305 atomic mass units (amu) and/or multiples of 305 to M^pro^, consistent with the addition of glutathione(s) via a disulfide bond. As revealed by RP-HPLC/MALDI-TOF MS analysis, treatment of 1.2 μM M^pro^ with 2 mM GSSG led to an estimated 45% monoglutathionylation (estimate based on the mass abundances), whereas treatment with 10 mM GSSG led to mono- (11%), di- (50%), and triglutathionylation (35%), with less than 4% of M^pro^ remaining unmodified (Fig. 1C). Comparing the results of Fig. 1A with those from Fig. 1C, the inhibition of M^pro^ activity closely correlated with the extent of glutathionylation. Although we found some variation in the extent of inhibition of M^pro^ by GSSG when using different preparations of M^pro^, the extent of inhibition always correlated with the extent of modification with glutathione. Interestingly, the data obtained with 2 mM GSSG suggested that modification of only one cysteine may be sufficient to lead to inhibition of M^pro^ activity, as this treatment yielded about 45% monoglutathionylation and little di- or triglutathionylation and showed an average 40% decrease in activity. By contrast, M^pro^ incubated at 18 μM during treatment with 2 mM GSSG revealed little reduction in activity and only small amounts of glutathionylation (Fig. 1B and D). Moreover, treatment of 18 μM M^pro^ with 10 mM GSSG led to only 14% monoglutathionylation (Fig. 1D), which was associated with an average inhibition of 18% (Fig. 1B), while no modification of M^pro^ occurred in the presence of 2 mM or 10 mM GSH (see Fig. S2A and S2B in the supplemental material).

10.1128/mBio.02094-21.3 FIG S1 Analysis of purified WT and C300S M^pro^ by SDS gel electrophoresis and RP-HPLC/MALDI-TOF MS analysis. (A) Amino acid sequence for WT M^pro^. The arrow indicates Cys300 which was mutated to Ser300 in the mutant M^pro^ (attempts to make Ala300 or Leu300 were unsuccessful). Shown below the sequence is the calculated molecular weight (M_r_) for WT and C300S M^pro^. (B and F) LDS gel electrophoresis of purified WT M^pro^ (B) and purified C300S M^pro^ (F). In each gel, lanes 1 and 4 contain molecular weight markers, while lanes 2 and 5 contain 4 μg and 8 μg of total protein loaded, respectively. (C and G) RP-HPLC showing the UV chromatogram at 205 nm for WT (C) and C300S (G) M^pro^. (D and H) The MALDI-TOF MS TIC chromatogram obtained by mass spectrometry for WT (D) and C300S (H) M^pro^. (E and I) Protein deconvolution of the peaks in panels D and H showing the determined molecular weight obtained for purified M^pro^ (33,797.5 experimental versus 33,796.5 calculated) (E) and C300S M^pro^ (33,781.8 experimental versus 33,780.4 calculated) (I) after protein deconvolution. The insets in panels E and I show the molecular ion profile used for deconvolution of each M^pro^. Separations were done on a Vydac C_18_ column using 95% buffer A (water with 0.1% formic acid and 0.02% trifluoroacetic acid) and 5% buffer B (acetonitrile with 0.1% formic acid and 0.02% trifluoroacetic acid) with a 0.5 ml min^−1^ flow rate and ramped to 65% buffer B with a 2% gradient for 30 min s and then ramped to 100% for the next 5 min and then returned to starting conditions 2 min later. M^pro^ eluted at approximately 25 min. The TOF settings were as follows: gas temperature, 350°C; drying gas rate, 13 liters/min; nebulizer, 55 pounds per square inch gauge (psig); sheath gas temperature, 350°C; fragmenter, 350 V; skimmer, 65 V. Molecular weights were determined by protein deconvolution using Agilent Mass Hunter software (Agilent). Download FIG S1, TIF file, 1.6 MB. Copyright © 2021 Davis et al. 2021 Davis et al. https://creativecommons.org/licenses/by/4.0/ This content is distributed under the terms of the Creative Commons Attribution 4.0 International license.

10.1128/mBio.02094-21.4 FIG S2 Exposure of SARS-CoV-2 M^pro^ to reduced glutathione does not lead to substantial glutathionylation of M^pro^. (A and B) M^pro^ molecular masses found by protein deconvolution for M^pro^ eluting off of the C_18_ reverse phase column following treatments with 2 and 10 mM reduced glutathione for 30 min for 1.2 μM M^pro^ (A) and 18 μM M^pro^ (B). The theoretical molecular mass of wild-type M^pro^ is 33,796.48. Download FIG S2, TIF file, 0.5 MB. Copyright © 2021 Davis et al. 2021 Davis et al. https://creativecommons.org/licenses/by/4.0/ This content is distributed under the terms of the Creative Commons Attribution 4.0 International license.

To better understand the nature of M^pro^ inhibition by glutathionylation, we modified 1.5 μM M^pro^ with 10 mM GSSG at pH 7.5 so that nearly all the M^pro^ was modified with at least one glutathione. Excess GSSG was removed by washing through an Amicon 10-kDa-cutoff membrane. RP-HPLC/MALDI-TOF MS analysis of this preparation on a C_18_ column followed by protein deconvolution indicated M^pro^ was now a mixture of mono- (23%), di- (68%), and triglutathionylated forms (9%) with little detectable unmodified M^pro^ (Fig. 1E). To determine whether the modification was reversible with thiol reducing agents, we treated glutathionylated M^pro^ with 10 mM dithiothreitol (DTT) for 30 min. This resulted in more than 90% of the glutathionylated M^pro^ being converted back to native M^pro^ (Fig. 1F). We then tested the activity of these preparations of M^pro^. Glutathionylated M^pro^ had less than 5% of the activity of unmodified M^pro^, confirming that glutathionylation was inhibiting protease activity (Fig. 1G). Following the addition of 10 mM DTT, the activity was fully restored, while DTT marginally improved native M^pro^ activity (Fig. 1G).

### Glutathionylation of M^pro^ inhibits M^pro^ dimerization.

**FIG 2:**
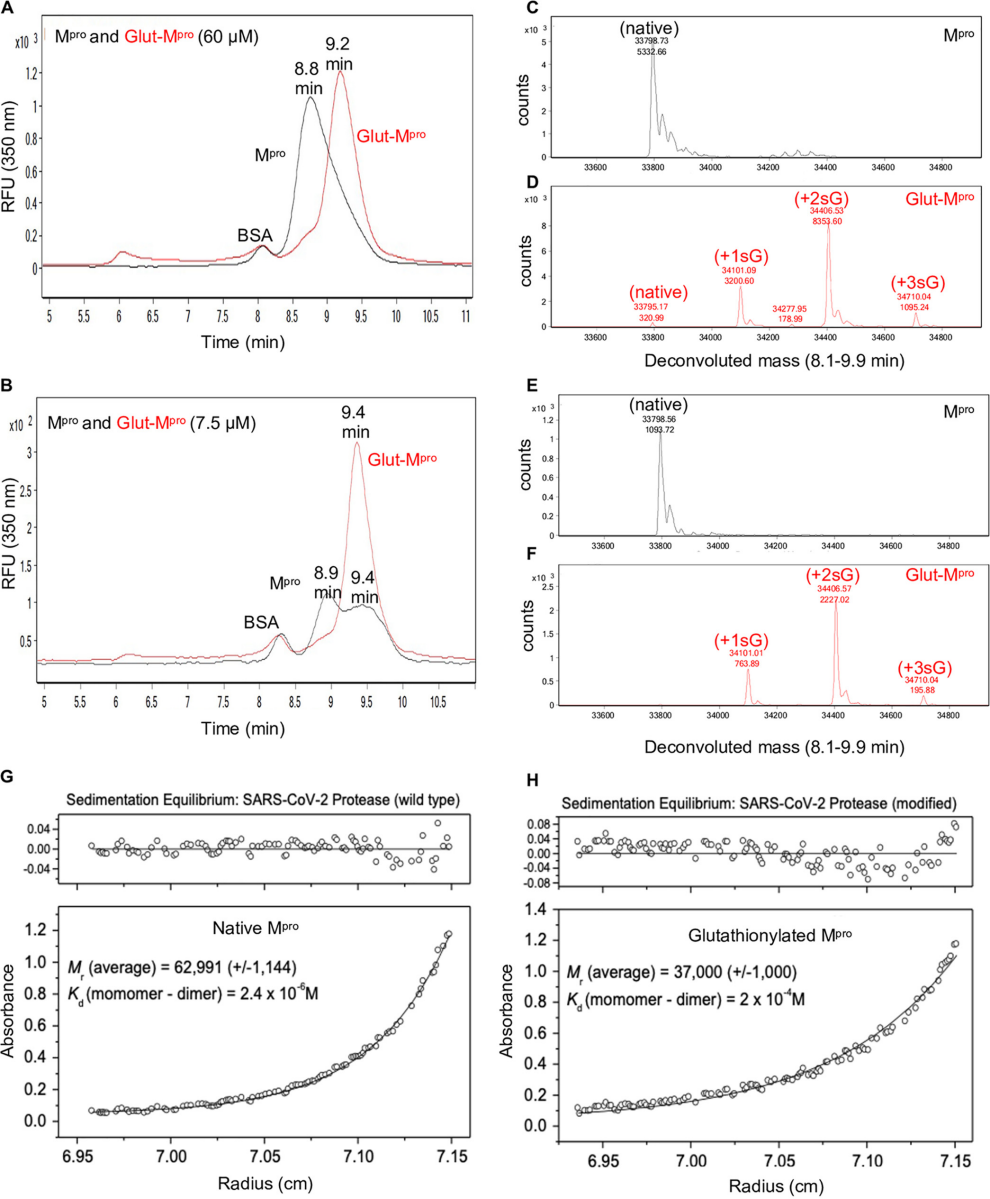
Glutathionylated M^pro^ behaves as a monomer based on size exclusion chromatography and equilibrium analytical ultracentrifugation. (A and B) M^pro^ and glutathionylated M^pro^ (Glut-M^pro^) were analyzed by SEC3000/MALDI-TOF MS, and the eluant was monitored by intrinsic protein fluorescence (in relative fluorescence units [RFU]) (excitation, 276 nm; emission, 350 nm). Glutathionylated M^pro^ was made with 10 mM GSSG at pH 7.5 for 2 to 2.5 h as described in Materials and Methods. (A and B) Overlay of the chromatograms for 60 μM (each) M^pro^ (black line) and glutathionylated M^pro^ (red line) (A) and 7.5 μM (each) M^pro^ (black line) and glutathionylated M^pro^ (red line) (B). (C and D) Protein deconvolution profiles for native M^pro^ (C) and glutathionylated M^pro^ (D) that were run as shown in panel A. (E and F) Protein deconvolution profile for native M^pro^ (E) and glutathionylated M^pro^ (F) that were run as shown in panel D. Shown above each peak are the molecular mass (top number) and the abundance (bottom number) found by protein deconvolution. The earlier eluting peak at 8.5 min is carboxymethylated BSA, which was used as a carrier in the runs of M^pro^ to help prevent nonspecific losses of M^pro^ during the run. (G and H) Equilibrium analytical ultracentrifugation of M^pro^ (G) and glutathionylated M^pro^ (H) (made as in panel A) at 0.6 mg ml^−1^ (18 μM) in 50 mM Tris buffer (pH 7.5), 2 mM EDTA, and 100 mM NaCl. The absorbance gradients in the centrifuge cell after the sedimentation equilibrium were attained at 21,000 rpm are shown in the bottom panels. The open circles represent the experimental values, and the solid lines represent the results of fitting to a single ideal species. The best fit for the data shown in panel G yielded a relative molecular weight (*M*_r_) of 62,991 ± 1,144 and a *K_d_* for dimerization of 2.4 μM, and that shown in panel H yielded a molecular weight of 37,000 ± 1,000 and a *K_d_* for dimerization of 200 μM. The corresponding top panels show the differences in the fitted and experimental values as a function of radial position (residuals). The residuals of these fits were random, indicating that the single species model is appropriate for the analyses.

To assess M^pro^ dimerization, we established a method consisting of size exclusion chromatography (SEC) coupled to mass spectrometry (MS) like that described previously for HIV-1 protease (12). We initially used SEC3000 columns and later SEC2000 columns from Phenomenex; both could be used successfully to separate M^pro^. When injected at 60 μM on a SEC3000 column, unmodified M^pro^ eluted at 8.8 min (Fig. 2A, black tracing), while glutathionylated M^pro^ eluted at 9.2 min (Fig. 2A, red tracing). When unmodified M^pro^ was injected at 7.5 μM, it clearly eluted as two peaks at 8.9 and 9.4 min consistent with a monomer-dimer behavior (Fig. 2B, black tracing), while the glutathionylated M^pro^ still eluted at 9.4 min consistent with a single species behavior (Fig. 2B, red tracing). Deconvolution of the eluting M^pro^ in Fig. 2A and B confirmed the expected masses for unmodified M^pro^ (Fig. 2C and E, black tracings) and the glutathionylated forms of M^pro^ (Fig. 2D and F, red tracings). Thus, the unmodified M^pro^ had a typical monomer/dimer two-species system running as dimers at high concentrations (60 μM) and as dimers and monomers at lower concentrations (7.5 μM). Dimerization of native M^pro^ was dependent on concentration, while glutathionylated M^pro^ behaved as a single monomer-like species independent of its concentration. Matched native and glutathionylated M^pro^ samples (18 μM) were analyzed by analytical ultracentrifugation (AUC) to obtain both the molecular mass of the species and the *K_d_* for dimerization. The results indicated that native M^pro^ was in equilibrium between monomeric and dimeric forms and behaved with a calculated dimerization *K_d_* of 2.4 μM (Fig. 2G), consistent with previous reports (6). At high concentrations (60 μM), M^pro^ was almost completely dimeric (Fig. 2G). By contrast, under the same conditions, the glutathionylated M^pro^ behaved almost completely monomeric with an estimated *K_d_* of 200 μM (Fig. 2H), indicating that glutathionylation was interfering with dimerization of M^pro^.

### Modification of a single cysteine of M^pro^ leads to inhibition of dimerization and activity.

**FIG 3:**
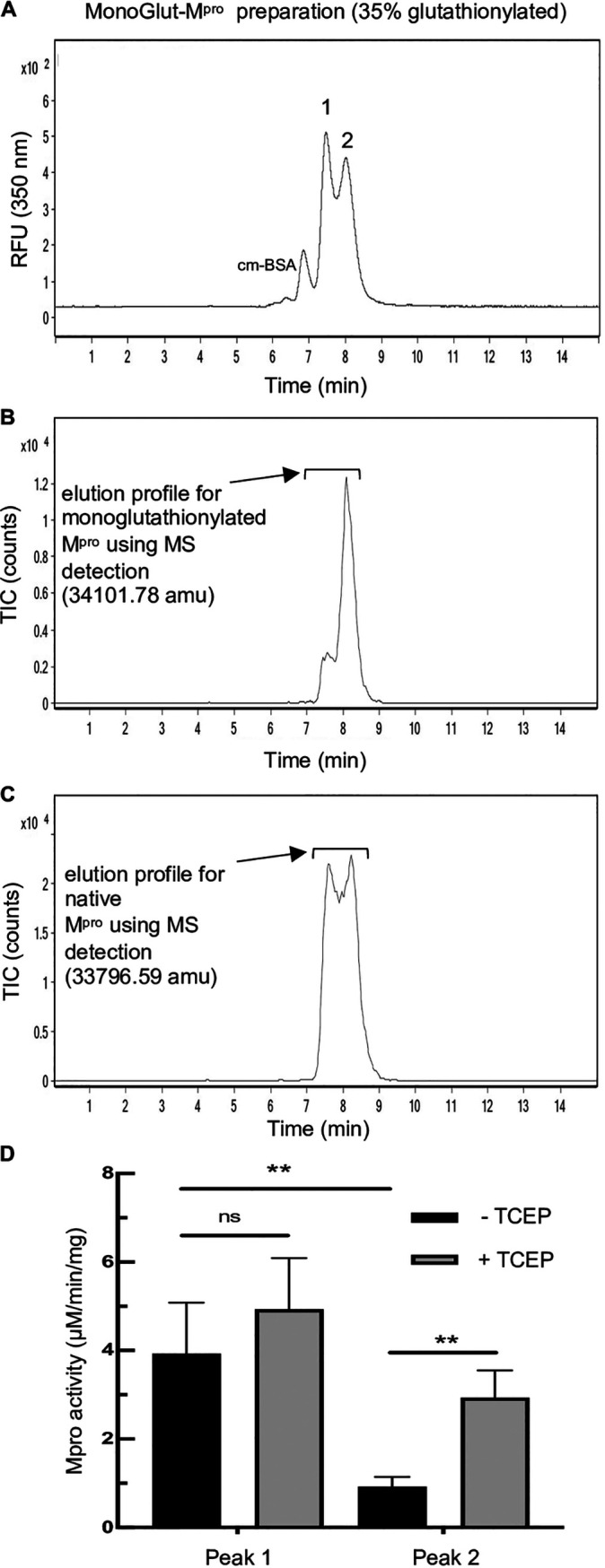
Monoglutathionylated M^pro^ has decreased activity and behaves as a monomer based on size exclusion chromatography. A preparation of M^pro^ containing a mixture of native and monoglutathionylated forms was made by incubating 1.2 μM M^pro^ with 5 mM GSSG for 2.5 h at 37°C at pH 6.8 , to predominantly modify the more reactive cysteines of M^pro^ as described in Materials and Methods. (A) SEC2000 elution profile as monitored using the intrinsic protein fluorescence (excitation, 276 nm; emission, 350 nm) of a 2-μl injection of 8 μM monoglutathionylated M^pro^ preparation. (B) SEC2000 elution profile of the same sample monitored using MALDI-TOF MS detection for the mass corresponding to the monoglutathionylated M^pro^ (34,101.78 amu). (C) SEC2000 elution profile of the same sample monitored using MALDI-TOF MS detection for the native M^pro^ mass (33,796.59 amu). (D) M^pro^ activity of peak 1 and peak 2 collected during the SEC separation of the monoglutathionylated M^pro^ preparation, without TCEP (black bars) and with TCEP treatment (gray bars) to remove the glutathione moiety. The values represent the average M^pro^ activity from four separate experiments (*n* = 4). **, *P* value of <0.01; ns, not significant (*P* value of  >0.05).

To determine whether glutathionylation of a single cysteine might render the enzyme monomeric and inactive, we generated a glutathionylated M^pro^ preparation by exposing 1.2 μM M^pro^ to 5 mM GSSG at pH 6.8, a pH that would favor the glutathionylation of only the most reactive cysteines (with low pK_a_s). This monoglutathionylated preparation had approximately 35% monoglutathionylated M^pro^ (see Fig. S3A in the supplemental material). We then analyzed the preparation (8 μM) by size exclusion and used MALDI-TOF MS detection to determine where the masses for monoglutathionylated and native M^pro^ eluted (using protein deconvolution). This glutathionylated preparation ran as two peaks consistent with the presence of both dimeric and monomeric forms of M^pro^ (Fig. 3A). Deconvolution of these two peaks revealed the elution profile for monoglutathionylated M^pro^ (Fig. 3B) and the elution profile for native M^pro^ (Fig. 3C). Interestingly, the mass for monoglutathionylated protease eluted from the size exclusion column predominantly (>70% of the total area) in the second peak, consistent with it behaving primarily as a monomer (Fig. 3B), while native M^pro^ eluted as both dimers and monomers as expected at this concentration (Fig. 3C and see Fig. 2B). Treatment of the glutathionylated M^pro^ preparation with reducing agent [Tris (2-carboxyethyl) phosphine hydrochloride (TCEP)] to remove the glutathione moiety led to an increase in the dimer peak and decrease in the monomer peak (see Fig. S3A and S3B in the supplemental material). Deconvolution revealed only the mass corresponding to native M^pro^ eluting across both dimeric and monomeric peaks (see Fig. S3B in the supplemental material). We also collected the first and second peaks eluting from SEC analysis of the monoglutathionylated preparation (peaks 1 and 2, labeled in Fig. 3A) and tested them for M^pro^ activity at equal protein concentrations. The activity of the second peak was only 25% of that of the first peak, consistent with monoglutathionylation causing the protease to elute primarily as a monomer and inhibiting M^pro^ activity (*P* < 0.01) (Fig. 3D). Treatment of the second peak with TCEP to remove the glutathione moiety, resulted in a significant increase in activity (*P* < 0.01) while having no significant effect (*P* > 0.05) on the activity of first peak (Fig. 3D). These data provide strong evidence that monoglutathionylated M^pro^ behaves as an inactive monomer and that dimerization potential and activity can be restored by removing the modification.

10.1128/mBio.02094-21.5 FIG S3 Deconvolution of the monoglutathionylated M^pro^ preparation before and after treatment with TCEP. A preparation of M^pro^ containing a mixture of native and monoglutathionylated forms was made by incubating 1.2 μM M^pro^ with 5 mM GSSG for 2.5 h at 37°C and pH 6.8 to increase specific modification of the more reactive cysteines of M^pro^ as described in Materials and Methods. (A) M^pro^ molecular weights found by protein deconvolution of the peaks in Fig. 3A, (B) M^pro^ molecular weights found by protein deconvolution of peaks in Fig. 3B after treatment with 50 mM TCEP for 15 min. Download FIG S3, TIF file, 0.6 MB. Copyright © 2021 Davis et al. 2021 Davis et al. https://creativecommons.org/licenses/by/4.0/ This content is distributed under the terms of the Creative Commons Attribution 4.0 International license.

### Inhibition of M^pro^ activity by glutathionylation is reversible with glutaredoxin (Grx).

**FIG 4:**
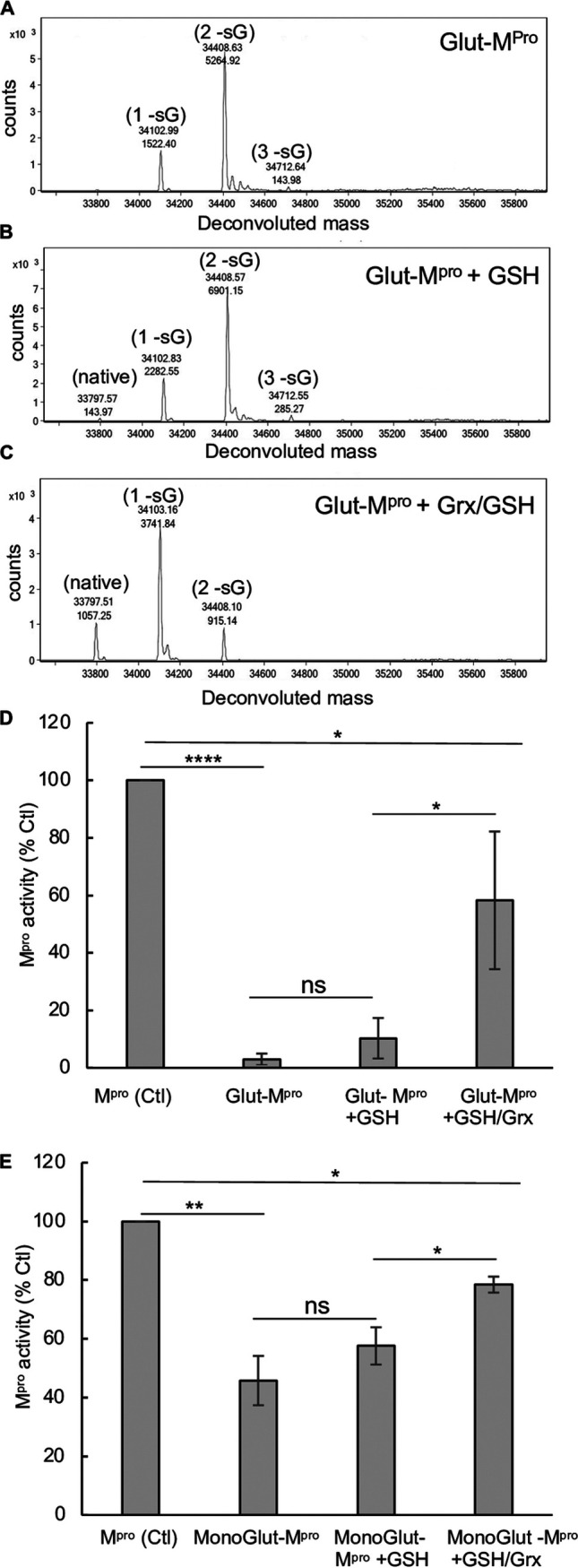
Grx deglutathionylates and restores activity of glutathionylated M^pro^. (A to C) M^pro^ glutathionylated at pH 7.5 was incubated (final concentration, 3 μM) for 30 min in the presence of buffer control (A), GSH (0.5 mM) (B), or GSH (0.5 mM) with Grx (final concentration, 350 nM) (C). Samples were analyzed by SEC3000/MALDI-TOF MS, and the eluting protease was analyzed by protein deconvolution (8.3 to 10 min) to determine the M^pro^ species present. The experimental masses (top number) are shown as well as the abundances (bottom number) for each peak obtained by deconvolution. The native M^pro^, as well as the increases in masses indicative of glutathionylation, are indicated for the addition of one (1 -sG), two (2 -sG), and three (3 -sG) glutathione moieties in the deconvolution profiles. (D) Samples of fully glutathionylated (<2% native) M^pro^ were treated as in panels A to C and then analyzed for M^pro^ activity (1 μM) using the RP-HPLC method for detection of pNa product (5 min) and compared to fully reduced (TCEP) treated glutathionylated M^pro^. M^pro^ activity for control in panel D was 5.77± 1.5 μM/min/mg, and percent activity for the different conditions was normalized to their fully reduced (TCEP) treated controls. (E) Samples of monoglutathionylated M^pro^ (7 μM total with approximately 3 μM monoglutathionylated) was incubated for 5 min in the presence of buffer, GSH (0.5 mM), or GSH (0.5 mM) with Grx (350 nM) and then analyzed for M^pro^ activity as indicated in panel D. The M^pro^ activity was normalized to TCEP-treated preparations which yielded fully reduced native M^pro^ and was used as 100% activity. For panels D and E, values represent the averages ± standard deviations of four and three separate experiments, respectively. Statistical significance with paired Student’s *t* test is indicated as follows: *, *P* value of <0.05; **, *P* value of  <0.01; ****, *P* value of <0.001; ns, not significant (*P* > 0.05).

Grx (also known as thioltransferase) is a ubiquitous cellular enzyme that can reverse glutathionylation of many cellular proteins. We tested whether Grx could deglutathionylate M^pro^ and restore its activity. Preparations of glutathionylated M^pro^ were prepared at pH 6.8 (to predominantly modify the most reactive cysteines) or pH 7.5 and tested for reversibility of glutathionylation and restoration of activity following treatment with Grx. The glutathionylated preparation made at pH 7.5 contained no detectable unmodified M^pro^ and was predominantly diglutathionylated M^pro^ (75%) and monoglutathionylated (22%) with the remainder triglutathionylated (3%) (Fig. 4A). Incubation of the preparation with 0.5 mM GSH alone, a cofactor required for Grx activity, produced a small amount of detectable unmodified M^pro^ (1.5%) and minor changes in percentages of other forms of M^pro^ (compare Fig. 4A with Fig. 4B). However, incubation of glutathionylated M^pro^ with Grx and 0.5 mM GSH resulted in loss of the triglutathionylated M^pro^, a substantial decrease in diglutathionylated M^pro^ (from 75% to 16%), and an increase in monoglutathionylated M^pro^ (22% to 65%) and native M^pro^ which made up 19% of the total M^pro^ (Fig. 4C). M^pro^ activity was then assessed under these same conditions. Incubation of glutathionylated M^pro^ with 350 nM Grx in the presence of 0.5 mM GSH led to a significant increase in protease activity, restoring an average 58% of the activity compared to untreated M^pro^, while 0.5 mM GSH alone restored only about 10% of the activity (Fig. 4D). We also assessed the ability of Grx to restore activity of the monoglutathionylated preparation made at pH 6.8. The glutathionylated preparation used in these experiments contained approximately 32% monoglutathionylated M^pro^ based on percent abundance and 4% diglutathionylated with the remainder (64%) unmodified. Incubation of this preparation of M^pro^ with 350 nm Grx with 0.5 mM GSH for just 5 min increased M^pro^ activity from 46% to 78% of control activity (Fig. 4E). GSH alone increased activity to a lesser degree from 46% to 58% of the control (Fig. 4E). In addition, Grx in a dose-dependent manner was able to deglutathionylate monoglutathionylated M^pro^ when incubated with only 0.1 mM GSH as assessed by SEC−MALDI-TOF MS and restore activity in a dose-dependent manner (see Fig. S4A to S4E in the supplemental material).

10.1128/mBio.02094-21.6 FIG S4 Grx reverses monoglutathionylation and restores M^pro^ activity. (A to C) Monoglutathionylated M^pro^ was incubated (8 μM final concentration) for 15 min in the presence of buffer control (A), GSH (0.1 mM) (B) or GSH (0.1 mM) with Grx (350 nM) (C), and samples were analyzed by SEC2000/MALDI-TOF MS deconvolution (7.3 to 8.6 min). (D and E) Samples were prepared as in panels A to C, and the percentage of monoglutathionylated M^pro^ and activity were determined after the 15-min incubation with 0, 88, 175, or 350 nm Grx in the presence of 100 μM GSH. (D and E) Percentage of monoglutathionylated Mpro after Grx treatment (D) and M^pro^ activity after Grx treatment (E). The M^pro^ activity was normalized to the TCEP-treated preparation which yielded fully reduced native M^pro^ and was used as 100% activity. For panel D, the values are the average of three separate experiments (*n* = 3), and for panel E, one experiment was performed in duplicate (*n* = 2). Download FIG S4, TIF file, 0.6 MB. Copyright © 2021 Davis et al. 2021 Davis et al. https://creativecommons.org/licenses/by/4.0/ This content is distributed under the terms of the Creative Commons Attribution 4.0 International license.

### Identification of glutathionylated cysteines by MALDI-TOF MS.

To determine which cysteines of M^pro^ are responsible for inhibition of dimerization and activity, we digested native M^pro^ and a monoglutathionylated preparation of M^pro^ (containing approximately 35% monoglutathionylated forms of M^pro^) with chymotrypsin or a combination of trypsin/lysC to produce peptides that could be assessed for glutathionylation. Prior to digestion, we alkylated free cysteines with *N*-ethylmaleimide (NEM) using the AccuMAP System (Promega); this step limits disulfide scrambling during alkylation and proteolytic digestion processes. From chymotrypsin digestions of native M^pro^ which was fully alkylated with NEM, we were able to identify alkylated peptides for 7 of the 12 cysteines of M^pro^, including cysteines 38, 44, 117, 128, 145, 156, and 300 by MS (see peptides 1 to 10 in Table S1 in the supplemental material) along with 12 other noncysteine peptides (see peptides 15 to 26 in Table S1 in the supplemental material). To identify which cysteines were becoming glutathionylated, we searched for predicted glutathionylated monoisotopic masses by molecular ion extraction of the total ion chromatogram (TIC) obtained from RP-HPLC/MALDI-TOF MS analysis of chymotrypsin digests. We located monoisotopic masses consistent with three glutathionylated peptides: ^151^NIDYDC^GSH^VSF^159^, ^295^DVVRQC^GSH^SGVTF^305^, and ^295^DVVRQC^GSH^SGVTFQ^306^ with glutathionylated Cys^156^, Cys^300^, and Cys^300^, respectively (Table 1; also see Fig. S5A to S5J for detailed analysis in the supplemental material). To confirm these peptides were, indeed, glutathionylated forms of the predicted M^pro^ native peptides, we analyzed the peptide digests after treatment with TCEP to remove disulfide-bound glutathione. When this was done, the masses for all three of the predicted glutathionylated peptides were no longer detected, due to the removal of glutathione with TCEP, and in its place we were able to locate the predicted native masses expected following removal of glutathione for all three peptides (Table 1 and see Fig. S5K to S5P in the supplemental material). These results demonstrated that Cys156 and Cys300 are both glutathionylated at pH 6.8 using GSSG modification (see Fig. S5 in the supplemental material).

**TABLE 1:** RP-HPLC/MALDI-TOF MS identification of peptides from chymotrypsin digestion of monoglutathionylated M^pro^ preparations without and with TCEP^*a*^

M^pro^ Cys	TCEP	Peptide^*b*^	*M*_r_		Δ	RT (min)
			Calc.	Expt.		
Cys156^*c*^	−	^151^NIDYDC^GSH^VSF^159^	1,379.50	1,379.47	0.03	19.0
Cys300	−	^295^DVVRQC^GSH^SGVTF^305^	1,514.66	1,514.62	0.04	14.9
Cys300^*d*^	−	^295^ DVVRQC ^GSH^ SGVTFQ ^306^	1,642.71	1,642.68	0.03	13.6
Cys156^*c*^	+	^151^ NIDYDCVSF ^159^	1,074.42	1,074.41	0.01	20.6
Cys300	+	^295^ DVVRQCSGVTF ^305^	1,209.58	1,209.56	0.02	16.9
Cys300^*d*^	+	^295^ DVVRQCSGVTFQ ^306^	1,337.63	1,337.61	0.02	15.4

a The retention times (RT) and molecular masses for the Cys300 peptides were confirmed with the use of synthetic peptides that were run on RP-HPLC/MALDI-TOF MS as native, alkylated, or glutathionylated peptides. Following chymotrypsin digestion, the peptide samples were analyzed in the absence (−) or presence (+) of 50 mM TCEP to remove glutathione moieties from the digested peptides. The calculated (Calc.) native masses (*M*_r_), the experimental (Expt.) masses, and the difference in mass (Δ) for each peptide are shown.

b GSH indicates modification of cysteine by glutathione based on a monoisotopic mass increase of approximately 305 amu (expected 305.08 amu).

c These peptides containing cysteine 156 occur due to lack of cleavage at the 154:155 predicted chymotryptic cleavage site.

d These peptides containing Cys300 occur due to incomplete cleavage at the 305:306 predicted chymotryptic cleavage site.

10.1128/mBio.02094-21.1 TABLE S1 RP-HPLC/MALDI-TOF MS identification of peptides after cysteine alkylation and chymotrypsin digestion of M^pro^ or monoglutathionylated M^pro^ preparations. Peptides 1 to 10 are the cysteine-containing peptides predicted and, where indicated, identified after chymotrypsin digestion. Peptides 11 to 14 are the glutathionylated and native forms of peptides identified. Peptides 16 to 27 are the non-cysteine-containing peptides predicted and, where indicated, identified after chymotrypsin digestion. “-sg” indicates glutathionylated peptide. **These peptides containing cysteine 156 occur due to lack of cleavage at the 154:155 predicted chymotryptic cleavage site. ***These peptides containing Cys300 occur due to incomplete cleavage at the 305:306 predicted chymotryptic cleavage site. ND, not detected. This is a work of the U.S. Government and is not subject to copyright protection in the United States. Foreign copyrights may apply. Download Table S1, PDF file, 0.03 MB. Copyright © 2021 Davis et al. 2021 Davis et al. https://creativecommons.org/licenses/by/4.0/ This content is distributed under the terms of the Creative Commons Attribution 4.0 International license.

10.1128/mBio.02094-21.7 FIG S5 Identification of glutathionylation at cysteines 156 and 300 in chymotryptic digests of monoglutathionylated M^pro^ preparations. The monoglutathionylated M^pro^ preparation was alkylated and digested with chymotrypsin as described in Materials and Methods and then analyzed by RP-HPLC/MALDI-TOF MS for the identification of M^pro^ peptides. (A and B) The relevant area of the RP-HPLC/MS TIC chromatogram for the elution of chymotryptic peptides before (A) or after (B) treatment of the digested peptide preparation with TCEP to remove glutathione from glutathionylated peptides. (C and D) The corresponding RP-HPLC/MS UV chromatogram at 205 nm for the elution of chymotryptic peptides before (C) or after (D) treatment of the digested peptide preparation with TCEP to remove glutathione from glutathionylated peptides. The arrows in panels A and C indicate the locations of eluting glutathionylated peptides 1^g^, 2^g^, and 3^g^, and in panels B and D, the arrows indicate the locations for the corresponding native peptides 1^n^, 2^n^, and 3^n^ detected after reduction with TCEP. “-SG” denotes a glutathionylated peptide. (E, G, and I) Identification of peak 3^g^ (E), peak 2^g^ (G), and peak 1^g^ (I) by ion extraction followed by monoisotopic deconvolution, revealing masses corresponding to the glutathionylated chymotryptic peptides: 151:159, 295:305, and 295:306. Each chromatogram shows the extracted ion chromatogram using the predicted monoisotopic masses [M + H]^+1^ for 151:159-SG, 295:305-SG, and 295:306-SG, respectively. (E, G, and I) The insets in panels E, G, and I show the deconvoluted monoisotopic masses obtained for the peak with the monoisotopic masses indicated by dashed arrows. (F, H, and J) The same analysis as in panels E, G, and I showing the absence of the glutathionylated peptides after TCEP reduction. (L, N, and P) Identification of peak 3^n^ (L), peak 2^n^ (N), and peak 1^n^ (P) by ion extraction followed by monoisotopic deconvolution revealing native peptides: 151:159, 295:305, and 295:306. Each chromatogram shows the extracted ion chromatogram using the predicted monoisotopic masses [M + H]^+1^ for 151:159, 295:305, and 295:306, respectively. The insets in panels L, N, and P show the deconvoluted monoisotopic masses obtained for each peptide with the monoisotopic masses indicated by dashed arrows. For panels K, M, and O, the same analysis is done on the TCEP-treated sample which reveals the loss of detection of the glutathionylated peptides. (L, N, and P) Identification of the native peptides 151:159 (L), 295:305 (N), and 295:306 (P) following TCEP treatment. Each panel shows the extracted ion chromatogram for the predicted monoisotopic masses 151:159, 295:305, and 295:306, respectively. The insets in panels L, N, and P show the deconvoluted monoisotopic masses obtained for each peptide with the monoisotopic masses indicated by dashed arrows. For panels K, M, and O, the same native molecular peptide ion extraction analysis is done on the non-TCEP-treated sample to show the absence of these peptides in the sample prior to TCEP treatment. Download FIG S5, TIF file, 1.4 MB. Copyright © 2021 Davis et al. 2021 Davis et al. https://creativecommons.org/licenses/by/4.0/ This content is distributed under the terms of the Creative Commons Attribution 4.0 International license.

Due to the inability to assess modification of cysteines 16, 22, 85, 161, and 265 using the chymotrypsin data, as the peptides carrying these residues were not located (see Table S1 for a list of the peptides found, in the supplemental material), we prepared trypsin/lysC digests of native M^pro^ and the same monoglutathionylated M^pro^ preparation used in chymotrypsin experiments. Using this approach, we were able to evaluate cysteines 16, 22, 85, and 265 (see Table S2 for a list of the peptides found, in the supplemental material). Interrogation of the TIC chromatogram for masses corresponding to glutathionylated forms of cysteine-containing peptides following tryspin/lysC digestion, revealed masses consistent with glutathionylation of three peptides: ^77^VIGHSMQNC^GSH^VLK^88^, ^299^QC^GSH^SGVTFQ^306^ and ^299^pyQC^GSH^SGVTFQ^306^ (the pyroglutamate (py) form of the 299-306 peptide which results from spontaneous deamidation of peptides with N-terminal glutamyl residues [23]) (Table 2 and see Fig. S6A to S6J in the supplemental material). These were glutathionylated at Cys^85^, Cys^300^, and Cys^300^, respectively (Table 2). Also, as with chymotrypsin digestion, the calculated masses for the three native forms were found following analysis of the tryptic digests after reduction with TCEP (Table 2 and see Fig. S6E to S6P in the supplemental material). The data from the trypsin/lysC digestion indicated that the majority of the monoglutathionylation was occurring at Cys300. We based this on the greater area at 205 nm obtained for glutathionylated Cys300 peptides than the Cys85 peptide (combined area for glutathionylated Cys300 peptides at 205 nm was 301 versus 56 for the glutathionylated Cys85 peptide) and their native forms (combined area at 205 nm for native Cys300 peptides was 272 versus 21 for the native Cys85 peptide) (see Fig. S6C and S6D in the supplemental material). Importantly, in all cases, the differences between the experimental and calculated peptide masses were less than 0.05 amu, providing strong confidence in their identity (Tables 1 and 2).

**TABLE 2:** RP-HPLC/MALDI-TOF MS identification of peptides from trypsin/lysC digestion of monoglutathionylated M^pro^ preparations without and with TCEP^*a*^

M^pro^ Cys	TCEP	Peptide^*b*^	*M*_r_		Δ	RT (min)
			Calc.	Expt.		
Cys85	−	^77^VIGHSMQNC^GSH^VLK^88^	1,632.74	1,632.71	0.03	13.5
Cys300	−	^299^QC^GSH^SGVTFQ^306^	1,173.44	1,173.42	0.02	10.9
Cys300^*c*^	−	^299^pyQC^GSH^SGVTFQ_306_	1,156.44	1,156.40	0.04	13.6
Cys85	+	^77^ VIGHSMQNCVLK ^88^	1,327.66	1,327.64	0.02	14.7
Cys300	+	^299^ QCSGVTFQ ^306^	868.36	868.36	0.00	11.2
Cys300^*c*^	+	^299^pyQCSGVTFQ^306^	851.36	851.33	0.03	14

a The retention times (RT) and molecular masses for the Cys300 peptides were confirmed with the use of synthetic peptides that were run on RP-HPLC/MALDI-TOF as native, alkylated, or glutathionylated peptides. Following trypsin/lysC digestion, the peptide samples were analyzed in the absence (**−**) or presence (+) of 50 mM TCEP to remove glutathione moieties from the digested peptides. The calculated (Calc.) native masses (*M*_r_), the experimental (Expt.) masses, and the difference in mass (Δ) for each peptide are shown.

b A GSH superscript after Cys indicates modification of cysteine by glutathione based on a monoisotopic mass increase of approximately 305 amu (expected 305.08 amu).

c These peptides are the result of the spontaneous deamidation that occurs with peptides containing an N-terminal glutamyl residues (23), and the retention times and molecular masses for this peptide were confirmed with the use of synthetic peptides that were run on RP-HPLC/MS.

10.1128/mBio.02094-21.2 TABLE S2 RP-HPLC/MALDI-TOF MS identification of peptides after cysteine alkylation and trypsin/lysC digestion of M^pro^ or monoglutathionylated M^pro^ preparation. Peptides 1 to 7 are the cysteine-containing peptides predicted and, where indicated, identified after trypsin/lysC digestion. Peptides 8 to 10 are the glutathionylated and native forms of peptides identified. Peptides 11 to 20 are the non-cysteine-containing peptides predicted and, where indicated, identified after trypsin/lysC digestion. “-sg” indicates glutathonylated peptide. **These peptides are the result of the spontaneous deamidation that occurs with peptides containing an N-terminal glutamine, and the retention times and molecular masses for this peptide were confirmed with the use of synthetic peptides that were run on RP-HPLC/MS. The retention times (RT) and molecular masses for the Cys300 peptides were confirmed with the use of synthetic peptides that were run on RP-HPLC/MALDI-TOF as native, alkylated, or glutathionylated peptides. Peptide samples were analyzed without (−) and with (+) TCEP to remove glutathione moieties. Shown are the calculated native masses [Mr(calc)] and the experimental masses [Mr(expt0)]. ND, not detected. This is a work of the U.S. Government and is not subject to copyright protection in the United States. Foreign copyrights may apply. Download Table S2, PDF file, 0.03 MB. Copyright © 2021 Davis et al. 2021 Davis et al. https://creativecommons.org/licenses/by/4.0/ This content is distributed under the terms of the Creative Commons Attribution 4.0 International license.

10.1128/mBio.02094-21.8 FIG S6 Identification of Cys300 as a major target for glutathionylation based on trypsin/lysC digests of monoglutathionylated M^pro^ preparations. The monoglutathionylated M^pro^ preparation was alkylated and digested with trypsin/lysC as described in Materials and Methods and then analyzed by RP-HPLC/MALDI-TOF MS for the identification of M^pro^ peptides. (A and B) The relevant area of the RP-HPLC/MS TIC chromatogram for the elution of trypsin/lysC generated peptides before (A) or after (B) treatment of the digested peptide preparation with TCEP to remove glutathione from glutathionylated peptides. (C and D) RP-HPLC/MS UV chromatogram at 205 nm for the elution of trypsin/lysC-generated peptides before (C) or after (D) treatment of the digested peptide preparation with TCEP to remove glutathione from glutathionylated peptides. The arrows in panels A and C indicate the locations of eluting glutathionylated peptides 1, 2, and 3, and in panels B and D, the arrows indicate the locations for the native peptides 1^n^, 2^n^, and 3^n^ detected after reduction with TCEP. “-SG” denotes glutathionylated peptide, and “(py)” denotes the pyroglutamate form of the 299-306 peptide that results from spontaneous deamidation of peptides with N-terminal glutamyl residues (23). (E to I) Detection and identification of glutathionylated typ/lysC peptides 77:88-SG, (G) 299:306-SG py (pyroglutamte form of the peptide) and (I) 295:306-SG peptides. Each panel in panels E, G, and I show the extracted ion chromatogram for the predicted monoisotopic masses for 77:88-SG, 299:306-SGpy, and 299:306-SG, respectively. The insets in panels E, G, and I show the deconvoluted monoisotopic masses obtained for each glutathionylated peptide with the monoisotopic masses indicated by dashed arrows. (F to J) The same analysis is done on the TCEP-treated sample which reveals the loss of detection of the glutathionylated peptides when carrying out the same mass extractions as in panels E, G, and I. (L, N, and P) Detection and identification of the native peptides 77:88 (L), 299:306py (N), and 295:306 (P) following TCEP treatment. Each panel shows the extracted ion chromatogram for the predicted monoisotopic masses for peptides 77:88, 299:306py, and 299:306, respectively. The insets in panels L, N, and P show the deconvoluted monoisotopic masses obtained for each peptide with the monoisotopic masses indicated by dashed arrows. For panels K, M and O, the same analysis is done on the untreated sample, which shows the absence of the peaks seen in panels L, N, and P prior to TCEP treatment. Download FIG S6, TIF file, 1.4 MB. Copyright © 2021 Davis et al. 2021 Davis et al. https://creativecommons.org/licenses/by/4.0/ This content is distributed under the terms of the Creative Commons Attribution 4.0 International license.

Together, the two approaches could identify peptides containing all cysteines of M^pro,^ except for Cys161. The combined data obtained from the chymotryptic and tryptic/lysC digestions of M^pro^ and glutathionylated M^pro^ showed that Cys85, Cys156, and Cys300 were glutathionylated. The trypsin/lysC 205-nm analysis provided evidence that only a minority of the glutathionylation was occurring at Cys85 (a similar analysis could not be done for Cys156 because of overlapping peaks in the chymotrypsin digests). Given the effects of glutathionylation on activity and dimerization and the importance of amino acids 298 and 299 for dimerization (4, 7), it suggested that Cys300, located at the dimer interface, is a primary target for glutathionylation of M^pro^ in its monomeric state and responsible for the effects we observed.

### Cys300 is required for inhibition of M^pro^ activity following glutathionylation.

**FIG 5:**
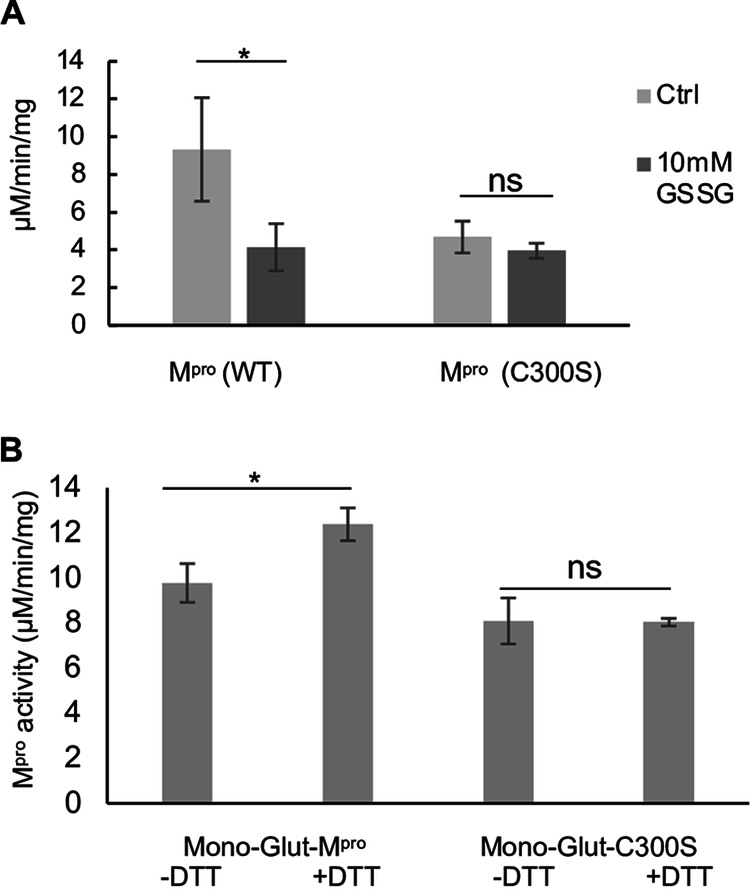
Glutathionylation inhibits wild-type (WT) SARS-Cov-2 M^pro^ activity but not C300S M^pro^ activity. (A) Activity of WT and C300S M^pro^ (1 μM enzyme) following 30-min preincubation of 1.2 μM M^pro^ with 10 mM oxidized glutathione. (B) M^pro^ activity for a WT monoglutathionylated M^pro^ preparation that had excess GSSG removed (containing approximately 30% monoglutathionylated M^pro^ and 4% diglutathionylated) and a C300S monoglutathionylated M^pro^ preparation (containing approximately 18% monoglutathionylated M^pro^) preincubated for 10 min without DTT (-DTT) or with 20 mM DTT (+DTT). The amount of monoglutathionylated M^pro^ was estimated using the relative abundances of native M^pro^ and glutathionylated M^pro^ following deconvolution of the eluting M^pro^ species from SEC/MALDI-TOF MS analysis. Values represent the averages ± standard deviations of three separate experiments (*n* = 3). Statistical significance: *, *P* value of <0.05; ns, not significant (*P* > 0.05).

To determine whether Cys300 was, in fact, the principal contributor to the inhibition of activity of M^pro^ following glutathionylation, we prepared a C300S mutant M^pro^ (for purity and molecular weight analysis, see Fig. S1F to S1I) and evaluated the effects of glutathionylation on M^pro^ activity. We noted that the basal activity of M^pro^ C300S was about 50% that of WT M^pro^. After 30 min of treatment at 1.2 μM with 10 mM GSSG, the activity of WT M^pro^ was inhibited by more than 50%. By contrast, this treatment did not affect the activity of C300S M^pro^ (Fig. 5A). We also measured the extent of glutathionylation for WT and C300S M^pro^ following the enzyme assay. On the basis of the absolute abundances of each form, we found that WT M^pro^ had 46%, 14%, and 5% mono-, di-, and triglutathionylated forms, respectively, with the remainder (35%) unmodified, while after the same treatment, C300S had 36% and 11% mono- and diglutathionylated forms, respectively, with the remainder (53%) unmodified. This indicated that while almost 50% of C300S could still become glutathionylated at other cysteine residues (possibly Cys85 and Cys156), its activity was unaffected, strongly implicating Cys300 in the inhibition of M^pro^ activity following glutathionylation of WT M^pro^. To determine whether Cys300 was the primary target for glutathionylation when incubating with GSSG at the lower pH of 6.8, we treated WT and C300S M^pro^ with 5 mM GSSG at pH 6.8 for 2.5 h to produce monoglutathionylated forms of M^pro^. Based on SEC/MALDI-TOF MS analysis, the WT M^pro^ was 36% glutathionylated, while the C300S M^pro^ was only 16% glutathionylated based on the abundances for each form (Fig. S7E and S7F). These data suggest that there are at least two reactive cysteines under these lower pH conditions. Activity of these preparations was measured before and after reduction with DTT. DTT increased the activity of the monoglutathionylated WT M^pro^ preparation by 26% but had no significant effect on the activity of monoglutathionylated C300S M^pro^ mutant (Fig. 5B). This suggests that while the C300S mutant can still become glutathionylated at alternative cysteines, the modification has little effect on M^pro^ activity.

GSSG is commonly used to probe the selective susceptibility of protein-cysteine residues to *S*-glutathionylation, as we have done here. However, to simulate intracellular oxidative stress conditions associated with viral infection, we also treated the M^pro^ protein with H_2_O_2_ in the presence of physiological relevant levels of GSH to represent the production of ROS in the normally reducing environment of the cell. We found that H_2_O_2_ in the presence of GSH promoted selective S-glutathionylation of WT-M^pro^ versus M^pro^-C300S, analogous to what we observed with GSSG (see Fig. S8A and S8B in the supplemental material).

**FIG 6:**
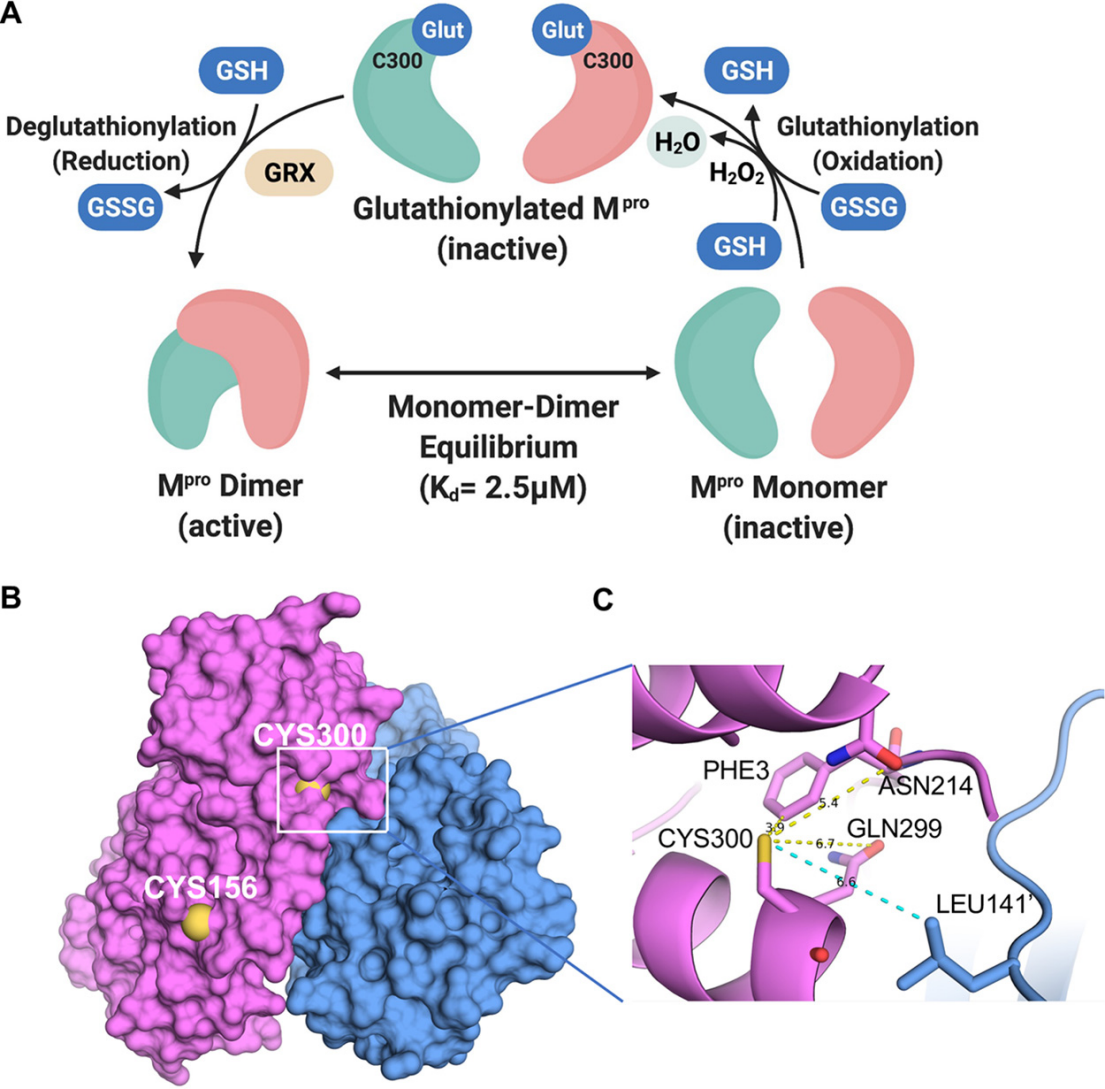
Model for the regulation of dimerization and activity through reversible glutathionylation of M^pro^ and space-filling and close-up ribbon model of SARS-CoV-2 M^pro^. (A) Model showing that M^pro^ dimer exists in equilibrium with its monomer form with a determined *K_d_* of 2.5 μM. The monomeric M^pro^ is susceptible to glutathionylation at Cys300, and this leads to inhibition of dimerization and loss of activity. Human Grx can reverse glutathionylation of Cys300 and restore dimerization and activity. (B) Space-filling model of the SARS-CoV-2 M^pro^ dimer (apo form) showing the location of cysteine 156 on the surface and cysteine 300 near the dimer interface in the left (magenta) protomer (PDB identifier [ID] 7K3T). (C) Close-up ribbon model around Cys300 showing the proximity to protomer 2 (blue) at leucine 141’ and the proximity to ASN214, GLN299, and PHE3 of protomer 1.

10.1128/mBio.02094-21.9 FIG S7 Glutathionylation of native and C300S M^pro^ after treatment with GSSG. Masses found by protein deconvolution from RP-HPLC/MALDI -TOF MS analysis of samples in Fig. 6A. (A and B) WT M^pro^ (A) or WT M^pro^ treated with 10 mM GSSG for 30 min (B). (C and D) Masses found by protein deconvolution for C300S M^pro^ (C) or C300S M^pro^ after treatment with 10 mM GSSG for 30 min (D). Samples were analyzed for protein glutathionylation following the assays performed in Fig. 6A. (E and F) SEC/MALDI-TOF analysis of WT (E) and C300S M^pro^ after glutathionylating at pH 6.8 with 5 mM GSSG as described in Materials and Methods (F). The upper number above each peak denotes the calculated mass, and the lower number denotes the abundance. Download FIG S7, TIF file, 0.6 MB. Copyright © 2021 Davis et al. 2021 Davis et al. https://creativecommons.org/licenses/by/4.0/ This content is distributed under the terms of the Creative Commons Attribution 4.0 International license.

10.1128/mBio.02094-21.10 FIG S8 Glutathionylation of native and C300S M^pro^ after treatment with GSH and H_2_O_2_ and comparison of the local environment around Cys300 in dimeric SARS-CoV-1 M^pro^ and dimeric SARS-CoV-2 M^pro^. (A) WT M^pro^ (squares) and C300S M^pro^ (circles) were incubated at 1.5 μM in the presence of 0.5 mM GSH in 50 mM Tris buffer (pH 7.0), 2 mM EDTA, and 300 mM NaCl, and treated with 0, 100, 250, 500, and 1,000 μM H_2_O_2_ for 15 min. The samples were then analyzed by SEC2000/MALDI-TOF MS to determine the percent glutathionylation of M^pro^ by protein deconvolution of the eluting M^pro^. The area for glutathionylated protease was divided by the total area (native and glutathionylated protease) and multiplied by 100 to obtain the percent glutathionylated protease. (B) Treatment of WT M^pro^ and C300S M^pro^ as in panel A using 250 μM H_2_O_2_. Values are averages ± standard deviations for three separate experiments. *, *P* value of <0.05. (C) Ball-and-stick model for local environment around Cys300 in SARS-CoV-1 M^pro^ showing the interactions with ASN214 and ASN299. M^pro^ (PDB ID 1UJ1) (SARS-CoV-1 M^pro^ apoenzyme at pH 6.0). (D) Ball-and-stick model for local environment around Cys300 in SARS-CoV-2 M^pro^ showing the interactions with ASN214 and ASN299 carbonyls (PDB ID 73KT) (SARS-CoV-2 M^pro^ apoenzyme at pH 6.5). Structural figures were produced with PyMOL v1.5.0.4 (40). Download FIG S8, TIF file, 0.7 MB. Copyright © 2021 Davis et al. 2021 Davis et al. https://creativecommons.org/licenses/by/4.0/ This content is distributed under the terms of the Creative Commons Attribution 4.0 International license.

## DISCUSSION

In cells that are under oxidative stress, cellular and foreign proteins may undergo glutathionylation, and this process, which is reversible, can alter the function of these proteins (20, 24–27). Some notable cellular proteins glutathionylated *in vivo* include Ras, beta-actin, IKK-beta, PTP1B, and caspase-3 (for reviews, see references 20 and 27). In this work, we found that dimerization and activity of M^pro^ can be regulated through reversible glutathionylation of Cys300 as depicted in our model in Fig. 6A. Cys85, Cys156, and Cys300 underwent measurable glutathionylation even at pH 6.8. However, only a minor amount of glutathionylation occurred at Cys85, and while Cys156 and Cys300 are both surface-exposed residues (Fig. 6B), we further investigated Cys300 due to its unique location at the dimer interface (Fig. 6B and C). Although there are numerous roles this regulatory system could play during SARS-CoV-2 replication in host cells under oxidative stress, it has nonetheless revealed a reactive cysteine that provides a novel target for the development of M^pro^ inhibitors that could be used to block SARS-CoV-2 replication.

A number of proteins have been shown to undergo glutathionylation in cells undergoing oxidative stress, and biochemical studies with GSSG, as performed here, can inform our understanding as to whether reversible glutathionylation might regulate the activity of key proteins (27). However, glutathionylation of proteins within cells undergoing oxidative stress is thought to more often go through sulfenic acid intermediates formed in the presence of H_2_O_2_ acting on susceptible thiols (27). Several reports have shown that studies in which proteins are exposed to 200 to 1000 μM H_2_O_2_ in the presence of GSH can provide insights on the glutathionylation of these proteins in cells undergoing oxidative stress (see, for example, references 28 and 29). In addition to the experiments performed with GSSG, we have also demonstrated that exposure of M^pro^ to GSH in the presence of physiologically relevant concentrations of H_2_O_2_ (28, 29) results in glutathionylation and that the degree of glutathionylation is about 50% less with C300S. With this background, the results here provide evidence to suggest that M^pro^ is reversibly glutathionylated in cells undergoing oxidative stress.

**FIG 7:**
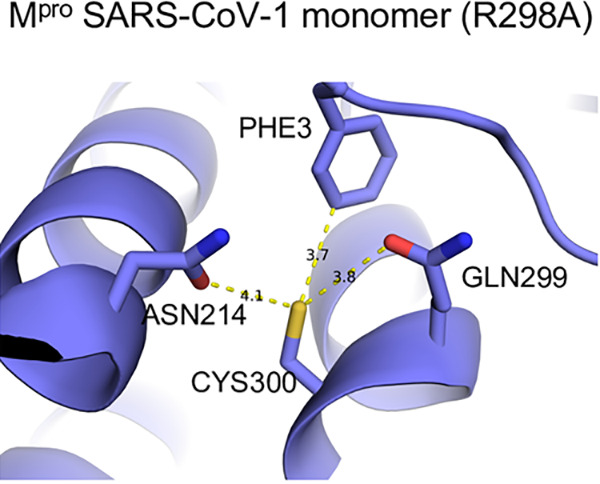
The local environment around Cys300 in monomeric SARS-CoV-1 M^pro^. Ball-and-stick model for local environment around Cys300 in R298A M^pro^ monomer PDB ID 2QCY (a monomeric form of SARS-CoV M^pro^ mutant R298A at pH 6.0). Structural figures were produced with PyMOL v1.5.0.4 (40).

Glutathionylation of proteins occurs via a mixed disulfide between glutathione and a cysteine residue. Most cysteine residues have relatively high pK_a_s (pH 8.0 or greater) and usually remain protonated under physiological conditions, making them relatively unreactive at typical cellular pH. However, studies have shown that the local environment around certain cysteine residues can lower their pK_a_, making them more susceptible to oxidation and glutathionylation (30–32). The local environment of Cys300 may account for this susceptibility to glutathionylation, and this provides a means to selectively target this cysteine residue with inhibitors. Previous studies have found that basic residues or serine hydroxyl sidechains in the local environment can substantially reduce the pK_a_ of thiols (30, 33). As for Cys300, there is a basic residue at Arg298 and a hydroxyl residue at Ser301. This may increase the local acidity of the Cys300 thiol group in the monomeric state, making it more prone to oxidation, while in the dimeric state, Arg298 is involved in interactions which stabilize the dimer (7). Inspection of a previously determined monomeric form of SARS-CoV-1 M^pro^ (R298A) reveals that the carbonyl side chains of Asn214 and Gln299, which can act as hydrogen acceptors and potentially destabilize the thiol group, show close contact with the Cys300 thiol (Fig. 7); this may enhance its reactivity. Although there is not a monomer structure of SARS-CoV-2 M^pro^, the distances of the Cys300 thiol to the carbonyls in SARS-CoV-1 and -2 dimer are much greater, possibly decreasing the reactivity of the dimeric form (see Fig. S8C and S8D in the supplemental material).

Why have SARS-CoV-2 and other RNA viruses evolved to have a dimeric protease susceptible to reversible inactivation by glutathionylation of a cysteine at the dimer interface? It is possible that this serves to blunt viral processing and replication in cells undergoing significant oxidative stress, which otherwise may generate defective viral particles (34). Also, high levels of M^pro^ are toxic to cells (35), and it is possible that this mechanism evolved to inactivate M^pro^ after cleavage by M^pro^ has released the viral proteins needed for replication as a result of virus-induced oxidative stress. Thus, Cys300 may act to regulate M^pro^ activity during viral replication to optimize the generation of new virions without prematurely killing the host cell. Moreover, M^pro^ from SARS-CoV-1 and SARS-CoV-2 contain 12 cysteines and 10 methionine residues. Studies have shown that such residues can also act as decoys to prevent permanent damage to proteins during oxidative stress (36, 37). In the case of M^pro^, this could help protect the active site cysteine required for catalysis. It should be noted that the details of the initial autocatalytic processing of M^pro^ from the polyproteins pp1a and pp1ab are still not fully understood, but in the case of HIV, we have shown that similar oxidative modifications can also reversibly inhibit the initial autocleavage of the Gag-Pol-Pro polyprotein, and this may similarly be true for autocleavage of M^pro^ from the polyproteins (8, 9, 11–13, 38). There is evidence that this may also be the case with Mason Pfizer monkey virus polyprotein processing (39).

It is interesting to speculate that this feature of coronavirus M^pro^ may have relevance to its evolution. M^pro^ from the three closest bat coronavirus relatives to SARS-CoV-2 (40) have an extremely high (>99%) degree of amino acid identity to that of SARS-CoV-2, and all three contain 12 cysteine residues, including Cys300. SARS-CoV-2 is thought to have jumped to humans from an original reservoir in *Rhinolophus* bats, possibly through an intermediate host (41). Bats are reservoirs for a vast number of coronaviruses and other RNA viruses and are often infected with these viruses without showing any signs of disease (42). One reason for this coexistence is that bats have evolved an immune response to RNA viruses with a minimal inflammatory response (42). In addition, the act of flying requires considerable metabolic energy, and when in flight and during migration, bats are placed under high levels of oxidative stress (43–45). Moreover, bats spend much of their lives in densely populated shelters such as caves that facilitate virus transmission. Not killing off the host bat colonies would appear to be a good evolutionary strategy for bat coronaviruses, and one can speculate that part of this evolutionary adaption might be dampening of viral replication under conditions of oxidative stress through the inhibition of M^pro^ by glutathionylation.

A more practical implication of our findings is that it can inform the development of antiviral drugs against SARS-CoV-2. While vaccines are effective at preventing COVID-19, effective anti-SARS-CoV-2 drugs are urgently needed and will remain so for the foreseeable future. Because of its essential role in SARS-CoV-2 replication, M^pro^ is an attractive target for drug development. Nearly all this effort has focused on active site inhibitors of M^pro^ which can block SARS-CoV-2 replication and cytopathic effect (6, 46–52). Although we carefully looked for glutathionylation of the active site cysteine, we could not confirm reversible glutathionylation occurring at Cys145. Our observation that Cys300 at the dimer interface is particularly susceptible to oxidative modification and that this modification can block dimerization of M^pro^, resulting in inhibition of activity, reveals an alternative way of targeting M^pro^. Being on the M^pro^ surface in the monomer, this cysteine may be highly accessible and may thus be a promising target for the development of specific M^pro^ inhibitors. In this regard, Günther and Reinke et al. (51) have recently identified the hydrophobic pocket consisting of Ile21, Leu253, Gln256, Val297, and Cys300 of SARS-Cov-2 M^pro^ as an allosteric binding site for two different compounds (51). Our results indicate that this area can be specifically targeted through Cys300, which is highly reactive and, if modified, leads to inhibition of dimerization.

## MATERIALS AND METHODS

### Enzymes, peptides, and reagents.

The substrate peptide for M^pro^ (H2N-TSAVLQ-pNA) and peptides corresponding to several predicted chymotryptic fragments containing cysteine residues, including M^pro^ peptides consisting of amino acids fragments 113:118, 127:134, 141:150, 155:159, 295:305 and 295:306 as well as the predicted tryptic fragment, 299:306, were obtained (>95% purity) from New England Peptide (Gardner, MA). Amicon ultracentrifugal filters (10-kDa cutoff, 0.5 ml, and 15 ml), carboxymethyl bovine serum albumin (cm-BSA), oxidized and reduced forms of l-glutathione (Bioxtra) (>98%), 4-nitroaniline (>99%), the reducing agents Tris (2-carboxyethyl) phosphine hydrochloride (TCEP) and dithiothreitol (DTT) were from Sigma-Aldrich (Milwaukee, WI). BioSep SEC3000 and SEC2000 size exclusion columns (300 × 4.6 mm) were from Phenomenex (Torrence, CA). The Vydac C_18_ column (218TP5205) was from MAC-MOD Analytical (Chadds Ford, PA). Peptide desalting columns from ThermoFisher Scientific (Pittsburgh, PA) and AccuMap low-pH protein digestion kit (with trypsin and lysC) and chymotrypsin (sequencing grade) were from Promega (Madison, WI). PreScisson protease was from GenScript (Piscataway, NJ). Recombinant human glutaredoxin (Grx) transcript variant 1 was from Origene (catalog no. TP319385) (Rockville, MD) and stored at −70°C in 25 mM Tris-HCl (pH 7.3), 100 mM glycine, and 10% glycerol (7 μM stock).

### Expression and purification of authentic M^pro^ and C300S M^pro^.

The SARS-CoV2 M^pro^-encoding sequence and C300S mutant sequence were cloned into pGEX-4T1 vector (Genscript) with N-terminal self-cleavage site (SAVLQ/SGFRK) and C-terminal His_6_ tag as previously designed by others (6). The plasmid constructs were transformed into BL21 Star (DE3) cells (ThermoFisher Scientific). The cultures were grown in terrific broth medium supplemented with ampicillin (Quality Biological, Gaithersburg, MD). At an optical density at 600 nm of 0.8, the cultures were induced by adding 1 mM isopropyl-α-d-thiogalactopyranoside and maintained at 20°C overnight. SARS-CoV2 M^pro^ and C300S M^pro^ were purified first by affinity chromatography using Talon cobalt-based affinity resin (TaKaRa Bio). The His_6_ tag was cleaved off by PreScission protease, and the resulting authentic 306-amino-acid M^pro^ (see Fig. S1A in the supplemental material) and C300S M^pro^ were further purified by SEC using a HiLoad Superdex 200 pg column (GE Healthcare) in 20 mM Tris (pH 7.5), 150 mM NaCl, and 2 mM DTT. The purity and molecular mass of M^pro^ were assessed by lithium dodecyl sulfate (LDS) gel electrophoresis as well as reverse-phase high-performance liquid chromatography (RP-HPLC) on a C_18_ column coupled with a matrix-assisted laser desorption ionization−time of flight (MALDI-TOF) mass spectrometer (MS). The purity of these M^pro^s was greater than 95% by LDS gel electrophoresis, RP-HPLC chromatography (205 nm), and MALDI-TOF MS analysis (see Fig. S1B to S1D and S1F to S1H in the supplemental material), with an average experimental mass of 33,796 amu ± 1 amu for WT (expected average mass of 33,796.48 amu) (see Fig. S1E and inset in the supplemental material) and an average experimental mass of 33,781.8 for C300S (expected average mass of 33,780.40 amu) (see Fig. S1I and inset in the supplemental material). The final preparations of M^pro^ (2 to 6 mg/ml) were stored at −70 in 40 mM Tris-HCl buffer (pH 7.5), 2 mM DTT, and 150 mM NaCl.

### M^pro^ colorimetric enzyme assay.

The enzymatic activity of M^pro^ of SARS-CoV-2 was measured using the custom-synthesized peptide H2N-TSAVLQ-pNA as described previously (21, 22). TSAVLQ represents the nsp4↓nsp5 cleavage sequence for SARS-CoV-1 and SARS-CoV-2 M^pro^. The rate of enzymatic activity was determined by following the increase in absorbance (390 nm) using a Spectramax 190 multiplate reader at 37°C as a function of time following the addition of substrate. Assays were conducted in clear flat bottom 96-well plates (Corning) in a total volume of 50 μl. Assays consisted of 40 μl of assay buffer (50 mM Tris [pH 7.5], 2 mM EDTA, and 300 mM NaCl containing 100 μg/ml of cm-BSA). Reactions were started by the addition of 10 μl of 2 mM substrate dissolved in ultrapure water and warmed to 37°C. Activity was obtained by measuring the increase in absorbance at 390 nm as a function of time within the linear range of the assay. A calibration curve was obtained for the product, 4-nitroanaline (pNA), and was used to convert the rate of the reaction to units of micromoles of product per minute per milligram of protein. In some cases, activity and M^pro^ modifications were determined by first stopping the assay at a set time (5 min) by acidification with formic acid (FA)/trifluoroacetic acid (TFA) and then analyzed by RP-HPLC using a 2% acetonitrile gradient on a Vydac C_18_ column as described below. The activity was calculated based on the amount of pNA product generated (detected at 390 nm) following RP-HPLC analysis.

### Glutathionylation of M^pro^ at pH 7.5 and pH 6.8.

To prepare glutathionylated M^pro^ for use in analytical ultracentrifugation, size exclusion chromatography (SEC), and activity assays, M^pro^ was first exchanged into a buffer containing 40 mM Tris-HCl, 2 mM EDTA, and 300 mM NaCl at pH 7.5 using Amicon 10-kDa-cutoff filter units. M^pro^ (1.5 μM) was then treated only with buffer or with a final concentration of 10 mM GSSG diluted from a stock of 200 mM GSSG that had been adjusted to neutral pH with sodium hydroxide. The solutions were then incubated at 37°C for 60 min or otherwise as described in Results before removing excess GSSG. To remove excess GSSG, the preparations were diluted 10× with buffer (50 mM Tris-HCl, 2 mM EDTA, and 100 mM NaCl) and washed four times using Amicon 10-kDa-cutoff filter units (0.5 ml). The final preparations were concentrated further with a 0.5-ml 10-kDa filtration unit (0.6 mg/ml). In some cases, these preparations were concentrated to 2 to 6 mg/ml for use in SEC. While the extent of glutathionylation varied among preparations of M^pro^, the procedure done at pH 7.5 usually yielded preparations of M^pro^ that contained predominantly diglutathionylated M^pro^ based on mass spectrometry (MS) deconvolution analysis as well as monoglutathionylated and triglutathionylated forms. To modify Mpro more selectively with GSSG, a similar procedure to that above was used except 5 mM GSSG was used and we lowered the buffer pH to 6.8. This pH was used to favor reaction of the most reactive cysteines (those cysteines whose pK_a_ may be lower than expected). Prior to modification, M^pro^ was treated with 50 mM Tris (2-carboxyethyl) phosphine hydrochloride (TCEP) for 30 min to ensure all cysteines were in their reduced form, and then TCEP was removed by multiple washes through an Amicon 10-kDa-cutoff filter with pH 6.8 incubation buffer (50 mM Tris-HCl, 2 mM EDTA, and 100 mM NaCl). For glutathionylation, M^pro^ (1.2 μM) was incubated for 2.5 h at 37°C in 50 mM Tris-HCl buffer, 300 mM NaCl, and 2 mM EDTA at pH 6.8 with either buffer only or 5 mM GSSG. The preparations were then washed four times to remove excess GSSG using Amicon 10-kDa-cutoff filter units (0.5 ml) with pH 6.8 buffer. This procedure typically resulted in 30 to 40% of M^pro^ becoming monoglutathionylated with less than 10% diglutathionylated. The percentage of the glutathionylated M^pro^ forms was estimated based on the abundances of the different protein forms (obtained by protein deconvolution). Although these modified forms of M^pro^ are not drastically different in molecular weight (differences of 305 and 610 amu, for example), they could have somewhat different ionization potentials, and therefore, the numbers are only an estimate of percent modification.

To confirm the identity of certain peptide fragments, we purchased synthetic peptides and modified them accordingly and determined their masses and retention times on the RP-HPLC/MS analysis. Peptides (100 μM) corresponding to chymotryptic fragments from digested M^pro^ (113:118, 127:134, 141:150, 155:159, and 295:305) were glutathionylated with 10 mM GSSG in 50 mM Tris-HCl buffer, 300 mM NaCl, and 2 mM EDTA (pH 7.5) for 1 h. These same peptides as well as 295:306 and the tryptic peptide 299:306 were alkylated with 5 mM *N*-ethylmaleimide (NEM) for 30 min at 37°C and then acidified to a pH of less than 3.0 with formic acid. Glutathionylation and NEM alkylation of the peptides was verified using RP-HPLC/TOF MS analysis on a Vydac C_18_ column with the same method that was used for analysis of trypsin/lysC and chymotrypsin digests of M^pro^ as described below.

### Grx assays on glutathionylated forms of M^pro^.

To determine whether Grx could deglutathionylate M^pro^, monoglutathionylated preparations of M^pro^ containing 30 to 40% monoglutathionylated or multiglutathionylated M^pro^ (prepared as described above in “Glutathionylation of M^pro^ at pH 7.5 and pH 6.8”) (8 μM) were used. For preparations made at pH 7.5 which had predominantly diglutathionylated M^pro^, the preparation was incubated at 37°C for 30 min in the presence of buffer control (50 mM Tris [pH 7.5], 2 mM EDTA, and 100 mM NaCl containing 100 μg/ml of carboxymethyl bovine serum albumin [cm-BSA]), Grx (350 nM) alone, GSH alone (0.5 mM), and Grx and GSH together. The samples were then analyzed for M^pro^ activity and by SEC3000/MALDI-TOF MS to assess the different forms of M^pro^. The eluting protease was analyzed by protein deconvolution (8.3 to 10 min) to determine the M^pro^ species present. Experiments with monoglutathionylated preparations made at pH 6.8 were also performed using GSH at 0.1 mM. In this case, the monoglutathionylated M^pro^ preparation was incubated for 15 min at 37°C in 50 mM Tris (pH 7.5), 2 mM EDTA, and 100 mM NaCl containing 100 μg/ml of cm-BSA, Grx (88 to 350 nM), 0.1 mM GSH, or 0.1 mM GSH with 88 to 350 nM Grx in a total volume of 10 μl. After incubation, an aliquot of each sample was assayed for M^pro^ activity (1 μM) and analyzed (2 μl) by SEC/MALDI-TOF MS to determine the percentage of glutathionylation in each treatment based on the abundances of each species. For these experiments, the enzyme activity was assessed after stopping the reactions by acidification with FA/TFA and determining the pNA product produced using RP-HPLC, as described above, to quantitate the amount of pNA product generated over the 5-min incubation. TCEP-treated glutathionylated enzyme was used to obtain the maximum native M^pro^ activity.

### Chymotrypsin and trypsin/lysC digestion and analysis of native and glutathionylated M^pro^.

Native M^pro^ and M^pro^ which was monoglutathionylated (∼30% to 40%) at pH 6.8 as described above was digested with chymotrypsin or trypsin/lysC using the Accumap low-pH sample preparation with urea under nonreducing conditions (Promega). The free cysteines in the M^pro^ preparations (100 μg) were first alkylated with *N*-ethylmaleimide in 8 M urea for 30 min at 37°C. Complete alkylation of all cysteines of the native M^pro^ with NEM was verified by RP-HPLC/TOF MS analysis. For chymotrypsin digestion, the alkylated proteins were diluted to 1 M urea with 100 mM Tris and 10 mM CaCl_2_ buffer (pH 8.0) (50 μg of protease in 57 μl added to 456 μl of buffer) and treated with 2.5 μg of chymotrypsin made fresh in 1 mM HCl. Samples were incubated overnight (18 h) at 37°C before stopping the reactions with a final concentration of 2% TFA to reach a pH of <3.0. For trypsin/recombinant lysC digestions, the alkylated proteins were digested with low-pH resistant lysC for 1 h at 37°C followed by continued digestion with AccuMAP modified trypsin and AccuMAP low-pH resistant lysC for 3 additional hours, as described in the AccuMAP protocol. The peptide digests were then desalted using peptide desalting columns (ThermoFisher) following the manufacturer’s instructions. The desalted clarified peptide mixtures were then dried in a Thermo speed vacuum system and resuspended in RP-HPLC solvent A (water with 0.1% FA and 0.02% TFA [0.1% FA/0.02% TFA]). Aliquots of the peptide digests were then analyzed without or with TCEP-Cl treatment (50 mM) to remove glutathione modifications and then were separated on a Vydac C_18_ column. For peptide analysis, the starting conditions were 100% solvent A (water with 0.1% FA/0.02% TFA). Elution of peptides was done with a 1%/min solvent B (acetonitrile with 0.1% FA/0.02% TFA) gradient over the first 20 min followed by a 2%/min gradient over the next 10 min. The elution of peptides was monitored using UV absorbance at 205, 254, and 276 nm as well as MALDI-TOF MS detection. Peptide digests were analyzed before and after TCEP treatment (for native M^pro^, see Fig. S6A and Fig. S6B for UV and TIC chromatograms, respectively; for monoglutathionylated M^pro^ digests without TCEP treatment, see Fig. S6C and Fig. S6D for UV and TIC chromatograms, respectively; for samples treated with TCEP, see Fig. S6E and Fig. S6F for UV and TIC chromatograms, respectively). Chymotrypsin digestion of alkylated M^pro^ is predicted to produce 10 alkylated cysteine-containing peptides in addition to 12 other non-cysteine-containing peptides of 3 amino acids or more. The predicted monoisotopic molecular masses for these peptides and their glutathionylated forms were used to extract specific peptide ions from the TIC chromatograms, and the masses found were further confirmed by monoisotopic deconvolution. When glutathionylated masses were found, we then searched for the native counterparts following TCEP reduction. We could locate 6 of the 10 predicted alkylated cysteine-containing peptides (covering 7 of the 12 cysteines) following chymotrypsin digestion of M^pro^ (see Table S1 for a list of peptides found in the supplemental material). In addition to the predicted cysteine-containing peptides, based on chymotrypsin digestion, the masses for two other cysteine-containing peptides were identified, including a 151:159 peptide fragment (containing Cys156) and a 305:306 peptide fragment (containing Cys300). These were produced, presumably, as a result of incomplete digestion by chymotrypsin at the 154:155 and 305:306 predicted cleavage sites (see Table S1, 7b and 10b, respectively, in the supplemental material). We also found molecular masses consistent with 10 other non-cysteine-containing peptides generated by chymotrypsin digestion (see Table S1 in the supplemental material).

Trypsin/lysC digests were analyzed by RP-HPLC/MALDI-TOF MS for both native (see Fig. S8A for TIC chromatogram and Fig. S8B for UV chromatogram in the supplemental material) and monoglutathionylated preparations before (see Fig. S8C for TIC chromatogram and Fig. S8D for UV chromatogram in the supplemental material) and after TCEP treatment. Trypsin/lysC digestion is predicted to yield seven cysteine-containing peptides and five of the seven cysteine alkylated peptides were found by molecular mass extraction from the TIC obtained by RP-HPLC/MALDI-TOF MS (see Table S2 in the supplemental material). In addition to the predicted cysteine-containing peptides, the masses for two other cysteine-containing peptides were identified including a 41:61 peptide, resulting from incomplete cleavage at the 60:61 trypsin cleavage site, and a mass consistent with the tryptic peptide 299:306 having undergone spontaneous formation of the pyroglutamate form of the peptide (see Table S2 in the supplemental material). This is commonly seen among peptides with N-terminal glutamates (23), and its retention time and mass were confirmed using a synthetic peptide standard that contained both the native and pyroglutamate forms of the peptide.

### RP-HPLC/TOF MS analysis.

Samples from the colorimetric enzyme assay, as described above, were analyzed by RP-HPLC with an Agilent 1200 series chromatograph on a Vydac C_18_ column (218TP5205, Hesperia, CA). Samples were injected (25 to 45 μl), and pNA substrate, pNA product, and native and modified forms of M^pro^ were eluted with a 2%/min acetonitrile gradient beginning with 95% solvent A (0.1% FA)/0.02% TFA) in RP-HPLC/MS grade water and 5% solvent B (0.1% FA/0.02% TFA in acetonitrile). The 2% gradient continued for 30 min and then was ramped to 95% acetonitrile for 2 min followed by a 5-min reequilibration to the starting conditions. Elution of samples was monitored at 320 nm (for pNA substrate) and 390 nm (for pNA product) with an Agilent diode array detector followed by MS analysis with an Agilent 6230 time of flight MS configured with Jetstream. M^pro^ and its glutathionylated forms eluted between 24 and 26 min (approximately 57% acetonitrile). The mass of the protein was determined by protein deconvolution using Agilent’s Mass Hunter software. The TOF settings were the following: gas temperature, 350°C; drying gas, 13 liters/min; nebulizer, 55 lb/in^2^; sheath gas temperature, 350°C; fragmentor, 145 V; skimmer, 65 V. The mass determination for peptides was done by deconvolution (resolved isotope) using Agilent Mass Hunter software (Agilent).

### Analysis of M^pro^ by SEC coupled with MALDI-TOF MS detection.

Size exclusion chromatography (SEC) on native and glutathionylated forms of M^pro^ was carried out using BioSep SEC3000 column and subsequently a BioSep SEC2000 column (300 mm × 4.6 mm; Phenomenex, Torrance, CA, USA) with 25 mM ammonium formate running buffer (pH 8.0) on a 1200 series HPLC-MS system (Agilent, Santa Clara, CA, USA). The isocratic flow rate was 0.35 ml · min^−1^, and M^pro^ samples were injected at 2 μl. Where indicated, cm-BSA was used as a carrier to help prevent nonspecific binding of protein during the analysis. Proteins eluting from the column were monitored using an Agilent 1100 series fluorescent detector connected in series with the Agilent 6230 TOF MS detector. At high concentrations, M^pro^ eluted as a single peak with a tailing edge, while at lower concentrations, M^pro^ eluted as two peaks consistent with it behaving as a monomer dimer system. For the SEC3000 column, the M^pro^ peaks eluted between 8.5 and 10 min, while for the SEC2000 column, peaks eluted between 7 and 8.5 min. The percentage of different forms of M^pro^ was estimated by using the abundances of each species which can provide only an estimate due to variations in ionization potential for each M^pro^ species.

### Analytical ultracentrifugation.

For analytical ultracentrifugation (AUC), a Beckman Optima XL-I analytical ultracentrifuge, with absorption optics, an An-60 Ti rotor, and standard double-sector centerpiece cells, was used. Sedimentation equilibrium measurements of authentic native M^pro^ and glutathionylated M^pro^ were used to determine the average molecular weight and dissociation constant (*K_d_*) for dimerization. M^pro^ was diluted into 50 mM Tris (pH 7.5) buffer containing 2 mM EDTA and 300 mM NaCl buffer to 1 μM (6-ml total solution) and then was left untreated or was glutathionylated with 10 mM GSSG for 45 min in the same buffer. Both preparations were washed by passing through a 10-kDa-cutoff Amicon membrane and washing 4 times with 50 mM Tris buffer with 2 mM EDTA and 100 mM NaCl. The preparations were analyzed by RP-HPLC/MS, and the control contained native M^pro^, while the glutathionylated preparation had predominantly diglutathionylated protease (63%), as well as triglutathionylated protease (22%) and monoglutathionylated protease (15%) based on their relative abundances. There was no detectable native M^pro^ remaining in this glutathionylated preparation. Proteins were concentrated to 0.6 mg/ml in 50 mM Tris buffer (pH 7.5) with 2 mM EDTA and 100 mM NaCl. Samples (100 μl) were centrifuged at 20°C at 21,000 rpm (16 h) and 45,000 (3 h) overspeed for baseline. Data (the average of 8 to 10 scans collected using a radial step size of 0.001 cm) were analyzed using the standard Optima XL-I data analysis software v6.03.

### Statistical analysis.

Statistical analyses were performed using two-tailed Student’s *t* test (paired) on experiments with at least three biological replicates. *P* values of >0.05 were not significant (ns) and values less or equal to 0.05 were considered statistically significant.

### Data availability.

All data are available in the main article or in the supplemental material.

## References

[1] Jin Z, Du X, Xu Y, Deng Y, Liu M, Zhao Y, Zhang B, Li X, Zhang L, Peng C, Duan Y, Yu J, Wang L, Yang K, Liu F, Jiang R, Yang X, You T, Liu X, Yang X, Bai F, Liu H, Liu X, Guddat LW, Xu W, Xiao G, Qin C, Shi Z, Jiang H, Rao Z, Yang H. 2020. Structure of M(pro) from SARS-CoV-2 and discovery of its inhibitors. Nature 582:289–293. doi:10.1038/s41586-020-2223-y. 32272481

[2] Hattori SI, Higshi-Kuwata N, Raghavaiah J, Das D, Bulut H, Davis DA, Takamatsu Y, Matsuda K, Takamune N, Kishimoto N, Okamura T, Misumi S, Yarchoan R, Maeda K, Ghosh AK, Mitsuya H. 2020. GRL-0920, an indole chloropyridinyl ester, completely blocks SARS-CoV-2 infection. mBio 11:e01833-20. doi:10.1128/mBio.01833-20. 32820005 PMC7441487

[3] Liang PH. 2006. Characterization and inhibition of SARS-coronavirus main protease. Curr Top Med Chem 6:361–376. doi:10.2174/156802606776287090. 16611148

[4] Xia B, Kang X. 2011. Activation and maturation of SARS-CoV main protease. Protein Cell 2:282–290. doi:10.1007/s13238-011-1034-1. 21533772 PMC4875205

[5] Anand K, Ziebuhr J, Wadhwani P, Mesters JR, Hilgenfeld R. 2003. Coronavirus main proteinase (3CLpro) structure: basis for design of anti-SARS drugs. Science 300:1763–1767. doi:10.1126/science.1085658. 12746549

[6] Zhang L, Lin D, Sun X, Curth U, Drosten C, Sauerhering L, Becker S, Rox K, Hilgenfeld R. 2020. Crystal structure of SARS-CoV-2 main protease provides a basis for design of improved alpha-ketoamide inhibitors. Science 368:409–412. doi:10.1126/science.abb3405. 32198291 PMC7164518

[7] Shi J, Sivaraman J, Song J. 2008. Mechanism for controlling the dimer-monomer switch and coupling dimerization to catalysis of the severe acute respiratory syndrome coronavirus 3C-like protease. J Virol 82:4620–4629. doi:10.1128/JVI.02680-07. 18305031 PMC2293028

[8] Davis DA, Brown CA, Newcomb FM, Boja ES, Fales HM, Kaufman J, Stahl SJ, Wingfield P, Yarchoan R. 2003. Reversible oxidative modification as a mechanism for regulating retroviral protease dimerization and activation. J Virol 77:3319–3325. doi:10.1128/jvi.77.5.3319-3325.2003. 12584357 PMC149757

[9] Davis DA, Dorsey K, Wingfield PT, Stahl SJ, Kaufman J, Fales HM, Levine RL. 1996. Regulation of HIV-1 protease activity through cysteine modification. Biochemistry 35:2482–2488. doi:10.1021/bi951525k. 8652592

[10] Davis DA, Newcomb FM, Moskovitz J, Wingfield PT, Stahl SJ, Kaufman J, Fales HM, Levine RL, Yarchoan R. 2000. HIV-2 protease is inactivated after oxidation at the dimer interface and activity can be partly restored with methionine sulphoxide reductase. Biochem J 346:305–311. doi:10.1042/0264-6021:3460305. 10677347 PMC1220854

[11] Davis DA, Yusa K, Gillim LA, Newcomb FM, Mitsuya H, Yarchoan R. 1999. Conserved cysteines of the human immunodeficiency virus type 1 protease are involved in regulation of polyprotein processing and viral maturation of immature virions. J Virol 73:1156–1164. doi:10.1128/JVI.73.2.1156-1164.1999. 9882317 PMC103936

[12] Davis DA, Tebbs IR, Daniels SI, Stahl SJ, Kaufman JD, Wingfield P, Bowman MJ, Chmielewski J, Yarchoan R. 2009. Analysis and characterization of dimerization inhibition of a multi-drug-resistant human immunodeficiency virus type 1 protease using a novel size-exclusion chromatographic approach. Biochem J 419:497–506. doi:10.1042/BJ20082068. 19149765 PMC2748811

[13] Davis DA, Newcomb FM, Starke DW, Ott DE, Mieyal JJ, Yarchoan R. 1997. Thioltransferase (glutaredoxin) is detected within HIV-1 and can regulate the activity of glutathionylated HIV-1 protease in vitro. J Biol Chem 272:25935–25940. doi:10.1074/jbc.272.41.25935. 9325327

[14] Davis DA, Newcomb FM, Moskovitz J, Fales HM, Levine RL, Yarchoan R. 2002. Reversible oxidation of HIV-2 protease. Methods Enzymol 348:249–259. doi:10.1016/s0076-6879(02)48643-0. 11885278

[15] Indukuri H, Castro SM, Liao SM, Feeney LA, Dorsch M, Coyle AJ, Garofalo RP, Brasier AR, Casola A. 2006. Ikkepsilon regulates viral-induced interferon regulatory factor-3 activation via a redox-sensitive pathway. Virology 353:155–165. doi:10.1016/j.virol.2006.05.022. 16806387

[16] Korenaga M, Wang T, Li Y, Showalter LA, Chan T, Sun J, Weinman SA. 2005. Hepatitis C virus core protein inhibits mitochondrial electron transport and increases reactive oxygen species (ROS) production. J Biol Chem 280:37481–37488. doi:10.1074/jbc.M506412200. 16150732

[17] Imai Y, Kuba K, Neely GG, Yaghubian-Malhami R, Perkmann T, van Loo G, Ermolaeva M, Veldhuizen R, Leung YH, Wang H, Liu H, Sun Y, Pasparakis M, Kopf M, Mech C, Bavari S, Peiris JS, Slutsky AS, Akira S, Hultqvist M, Holmdahl R, Nicholls J, Jiang C, Binder CJ, Penninger JM. 2008. Identification of oxidative stress and Toll-like receptor 4 signaling as a key pathway of acute lung injury. Cell 133:235–249. doi:10.1016/j.cell.2008.02.043. 18423196 PMC7112336

[18] Soucy-Faulkner A, Mukawera E, Fink K, Martel A, Jouan L, Nzengue Y, Lamarre D, Vande Velde C, Grandvaux N. 2010. Requirement of NOX2 and reactive oxygen species for efficient RIG-I-mediated antiviral response through regulation of MAVS expression. PLoS Pathog 6:e1000930. doi:10.1371/journal.ppat.1000930. 20532218 PMC2880583

[19] Suhail S, Zajac J, Fossum C, Lowater H, McCracken C, Severson N, Laatsch B, Narkiewicz-Jodko A, Johnson B, Liebau J, Bhattacharyya S, Hati S. 2020. Role of oxidative stress on SARS-CoV (SARS) and SARS-CoV-2 (COVID-19) infection: a review. Protein J 39:644–656. doi:10.1007/s10930-020-09935-8. 33106987 PMC7587547

[20] Shelton MD, Mieyal JJ. 2008. Regulation by reversible S-glutathionylation: molecular targets implicated in inflammatory diseases. Mol Cells 25:332–346. 18483468 PMC3367451

[21] Huang C, Wei P, Fan K, Liu Y, Lai L. 2004. 3C-like proteinase from SARS coronavirus catalyzes substrate hydrolysis by a general base mechanism. Biochemistry 43:4568–4574. doi:10.1021/bi036022q. 15078103

[22] Wei P, Fan K, Chen H, Ma L, Huang C, Tan L, Xi D, Li C, Liu Y, Cao A, Lai L. 2006. The N-terminal octapeptide acts as a dimerization inhibitor of SARS coronavirus 3C-like proteinase. Biochem Biophys Res Commun 339:865–872. doi:10.1016/j.bbrc.2005.11.102. 16329994 PMC7092940

[23] Wright HT. 1991. Nonenzymatic deamidation of asparaginyl and glutaminyl residues in proteins. Crit Rev Biochem Mol Biol 26:1–52. doi:10.3109/10409239109081719. 1678690

[24] Huang Z, Pinto JT, Deng H, Richie JP, Jr. 2008. Inhibition of caspase-3 activity and activation by protein glutathionylation. Biochem Pharmacol 75:2234–2244. doi:10.1016/j.bcp.2008.02.026. 18395187 PMC2453044

[25] Cabiscol E, Levine RL. 1996. The phosphatase activity of carbonic anhydrase III is reversibly regulated by glutathiolation. Proc Natl Acad Sci USA 93:4170–4174. doi:10.1073/pnas.93.9.4170. 8633035 PMC39506

[26] Mieyal JJ, Chock PB. 2012. Posttranslational modification of cysteine in redox signaling and oxidative stress: focus on S-glutathionylation. Antioxid Redox Signal 16:471–475. doi:10.1089/ars.2011.4454. 22136616 PMC3270050

[27] Mieyal JJ, Gallogly MM, Qanungo S, Sabens EA, Shelton MD. 2008. Molecular mechanisms and clinical implications of reversible protein S-glutathionylation. Antioxid Redox Signal 10:1941–1988. doi:10.1089/ars.2008.2089. 18774901 PMC2774718

[28] Zmijewski JW, Banerjee S, Bae H, Friggeri A, Lazarowski ER, Abraham E. 2010. Exposure to hydrogen peroxide induces oxidation and activation of AMP-activated protein kinase. J Biol Chem 285:33154–33164. doi:10.1074/jbc.M110.143685. 20729205 PMC2963401

[29] Lamberts RR, Onderwater G, Hamdani N, Vreden MJ, Steenhuisen J, Eringa EC, Loer SA, Stienen GJ, Bouwman RA. 2009. Reactive oxygen species-induced stimulation of 5'AMP-activated protein kinase mediates sevoflurane-induced cardioprotection. Circulation 120:S10–S15. doi:10.1161/CIRCULATIONAHA.108.828426. 19752353

[30] Naor MM, Jensen JH. 2004. Determinants of cysteine pKa values in creatine kinase and alpha1-antitrypsin. Proteins 57:799–803. doi:10.1002/prot.20261. 15476207

[31] D’Ettorre C, Levine RL. 1994. Reactivity of cysteine-67 of the human immunodeficiency virus-1 protease: studies on a peptide spanning residues 59 to 75. Arch Biochem Biophys 313:71–76. doi:10.1006/abbi.1994.1360. 8053689

[32] Karlstrom AR, Shames BD, Levine RL. 1993. Reactivity of cysteine residues in the protease from human immunodeficiency virus: identification of a surface-exposed region which affects enzyme function. Arch Biochem Biophys 304:163–169. doi:10.1006/abbi.1993.1334. 8323281

[33] Awoonor-Williams E, Rowley CN. 2016. Evaluation of methods for the calculation of the pKa of cysteine residues in proteins. J Chem Theory Comput 12:4662–4673. doi:10.1021/acs.jctc.6b00631. 27541839

[34] Ciriolo MR, Palamara AT, Incerpi S, Lafavia E, Bue MC, De Vito P, Garaci E, Rotilio G. 1997. Loss of GSH, oxidative stress, and decrease of intracellular pH as sequential steps in viral infection. J Biol Chem 272:2700–2708. doi:10.1074/jbc.272.5.2700. 9006907

[35] Resnick SJ, Iketani S, Hong SJ, Zask A, Liu H, Kim S, Melore S, Nair MS, Huang Y, Tay NES, Rovis T, Yang HW, Stockwell BR, Ho DD, Chavez A. 29 August 2020. A simplified cell-based assay to identify coronavirus 3CL protease inhibitors. bioRxiv 10.1101/2020.08.29.272864.

[36] Luo S, Levine RL. 2009. Methionine in proteins defends against oxidative stress. FASEB J 23:464–472. doi:10.1096/fj.08-118414. 18845767 PMC2630790

[37] Requejo R, Hurd TR, Costa NJ, Murphy MP. 2010. Cysteine residues exposed on protein surfaces are the dominant intramitochondrial thiol and may protect against oxidative damage. FEBS J 277:1465–1480. doi:10.1111/j.1742-4658.2010.07576.x. 20148960 PMC2847196

[38] Daniels SI, Davis DA, Soule EE, Stahl SJ, Tebbs IR, Wingfield P, Yarchoan R. 2010. The initial step in human immunodeficiency virus type 1 GagProPol processing can be regulated by reversible oxidation. PLoS One 5:e13595. doi:10.1371/journal.pone.0013595. 21042582 PMC2962637

[39] Parker SD, Hunter E. 2001. Activation of the Mason-Pfizer monkey virus protease within immature capsids in vitro. Proc Natl Acad Sci USA 98:14631–14636. doi:10.1073/pnas.251460998. 11724937 PMC64733

[40] Jaimes JA, Andre NM, Chappie JS, Millet JK, Whittaker GR. 2020. Phylogenetic analysis and structural modeling of SARS-CoV-2 spike protein reveals an evolutionary distinct and proteolytically sensitive activation loop. J Mol Biol 432:3309–3325. doi:10.1016/j.jmb.2020.04.009. 32320687 PMC7166309

[41] Andersen KG, Rambaut A, Lipkin WI, Holmes EC, Garry RF. 2020. The proximal origin of SARS-CoV-2. Nat Med 26:450–452. doi:10.1038/s41591-020-0820-9. 32284615 PMC7095063

[42] Banerjee A, Baker ML, Kulcsar K, Misra V, Plowright R, Mossman K. 2020. Novel insights into immune systems of bats. Front Immunol 11:26. doi:10.3389/fimmu.2020.00026. 32117225 PMC7025585

[43] Wilhelm Filho D, Althoff SL, Dafre AL, Boveris A. 2007. Antioxidant defenses, longevity and ecophysiology of South American bats. Comp Biochem Physiol C Toxicol Pharmacol 146:214–220. doi:10.1016/j.cbpc.2006.11.015. 17257902

[44] Chionh YT, Cui J, Koh J, Mendenhall IH, Ng JHJ, Low D, Itahana K, Irving AT, Wang LF. 2019. High basal heat-shock protein expression in bats confers resistance to cellular heat/oxidative stress. Cell Stress Chaperones 24:835–849. doi:10.1007/s12192-019-01013-y. 31230214 PMC6629734

[45] Costantini D, Lindecke O, Pētersons G, Voigt CC. 2019. Migratory flight imposes oxidative stress in bats. Curr Zool 65:147–153. doi:10.1093/cz/zoy039. 30936903 PMC6430974

[46] Hattori SI, Higashi-Kuwata N, Hayashi H, Allu SR, Raghavaiah J, Bulut H, Das D, Anson BJ, Lendy EK, Takamatsu Y, Takamune N, Kishimoto N, Murayama K, Hasegawa K, Li M, Davis DA, Kodama EN, Yarchoan R, Wlodawer A, Misumi S, Mesecar AD, Ghosh AK, Mitsuya H. 2021. A small molecule compound with an indole moiety inhibits the main protease of SARS-CoV-2 and blocks virus replication. Nat Commun 12:668. doi:10.1038/s41467-021-20900-6. 33510133 PMC7843602

[47] Amin SA, Banerjee S, Ghosh K, Gayen S, Jha T. 2021. Protease targeted COVID-19 drug discovery and its challenges: insight into viral main protease (Mpro) and papain-like protease (PLpro) inhibitors. Bioorg Med Chem 29:115860. doi:10.1016/j.bmc.2020.115860. 33191083 PMC7647411

[48] Banerjee R, Perera L, Tillekeratne LMV. 2021. Potential SARS-CoV-2 main protease inhibitors. Drug Discov Today 26:804–816. doi:10.1016/j.drudis.2020.12.005. 33309533 PMC7724992

[49] Capasso C, Nocentini A, Supuran CT. 2021. Protease inhibitors targeting the main protease and papain-like protease of coronaviruses. Expert Opin Ther Pat 31:309–324. doi:10.1080/13543776.2021.1857726. 33246378

[50] Cui W, Yang K, Yang H. 2020. Recent progress in the drug development targeting SARS-CoV-2 main protease as treatment for COVID-19. Front Mol Biosci 7:616341. doi:10.3389/fmolb.2020.616341. 33344509 PMC7746807

[51] Günther S, Reinke PYA, Fernández-García Y, Lieske J, Lane TJ, Ginn HM, Koua FHM, Ehrt C, Ewert W, Oberthuer D, Yefanov O, Meier S, Lorenzen K, Krichel B, Kopicki JD, Gelisio L, Brehm W, Dunkel I, Seychell B, Gieseler H, Norton-Baker B, Escudero-Pérez B, Domaracky M, Saouane S, Tolstikova A, White TA, Hänle A, Groessler M, Fleckenstein H, Trost F, Galchenkova M, Gevorkov Y, Li C, Awel S, Peck A, Barthelmess M, Schlünzen F, Lourdu Xavier P, Werner N, Andaleeb H, Ullah N, Falke S, Srinivasan V, França BA, Schwinzer M, Brognaro H, Rogers C, Melo D, Zaitseva-Doyle JJ, Knoska J, Peña-Murillo GE, et al. 2021. X-ray screening identifies active site and allosteric inhibitors of SARS-CoV-2 main protease. Science 372:642−646. doi:10.1126/science.abf7945. 33811162 PMC8224385

[52] Jin Z, Wang H, Duan Y, Yang H. 2021. The main protease and RNA-dependent RNA polymerase are two prime targets for SARS-CoV-2. Biochem Biophys Res Commun 538:63–71. doi:10.1016/j.bbrc.2020.10.091. 33288200 PMC7680044

