# Supplementary figures and images for: Regulation of the Dimerization and Activity of SARS-CoV-2 Main Protease through Reversible Glutathionylation of Cysteine 300
Source: mBio. 2021 Aug 17;12(4):e02094-21. doi: 10.1128/mBio.02094-21 (PMC8406260; doi:10.1128/mBio.02094-21)

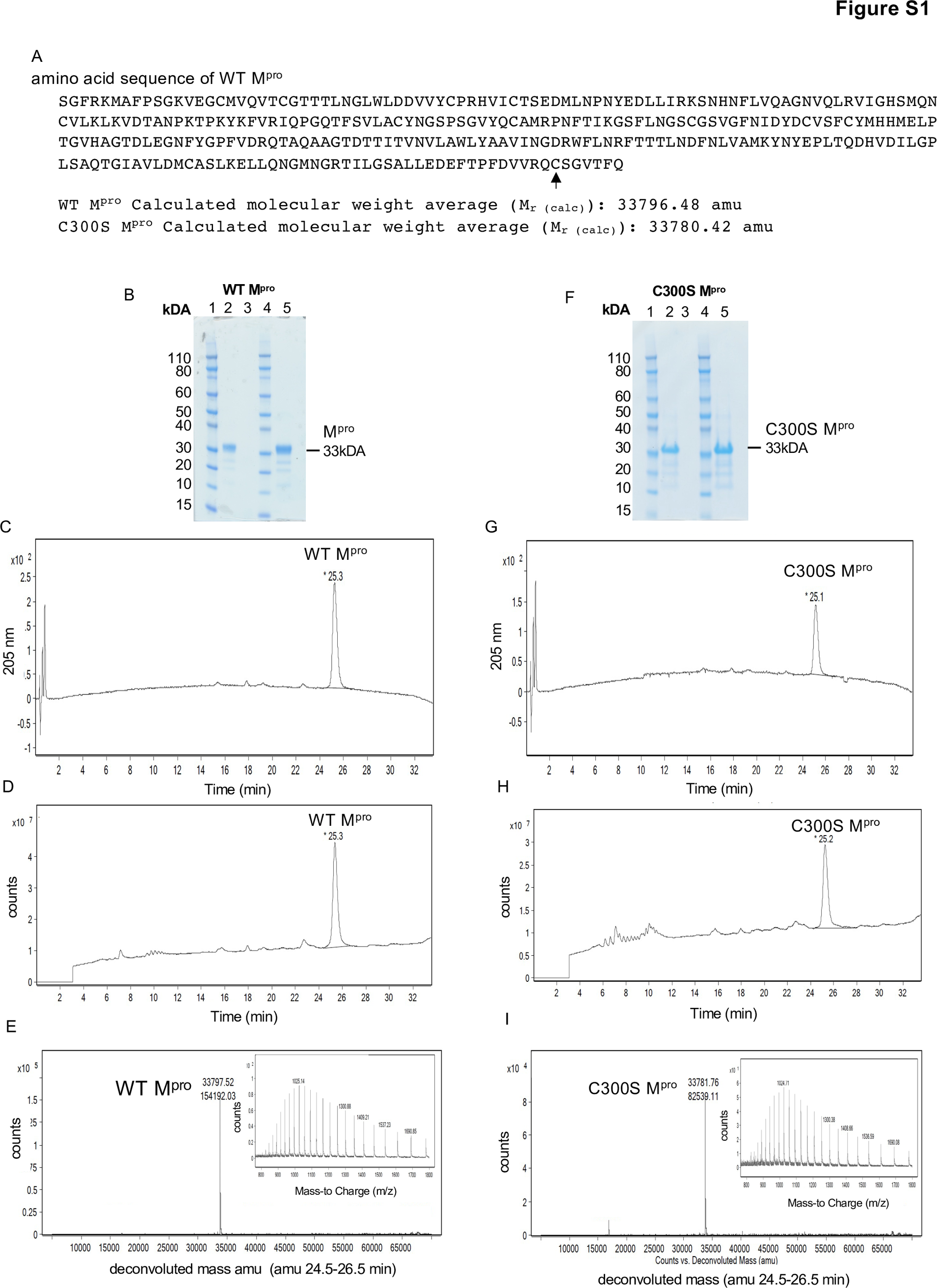

Supplement: FIG S1 [file mbio.02094-21-sf001.tif]

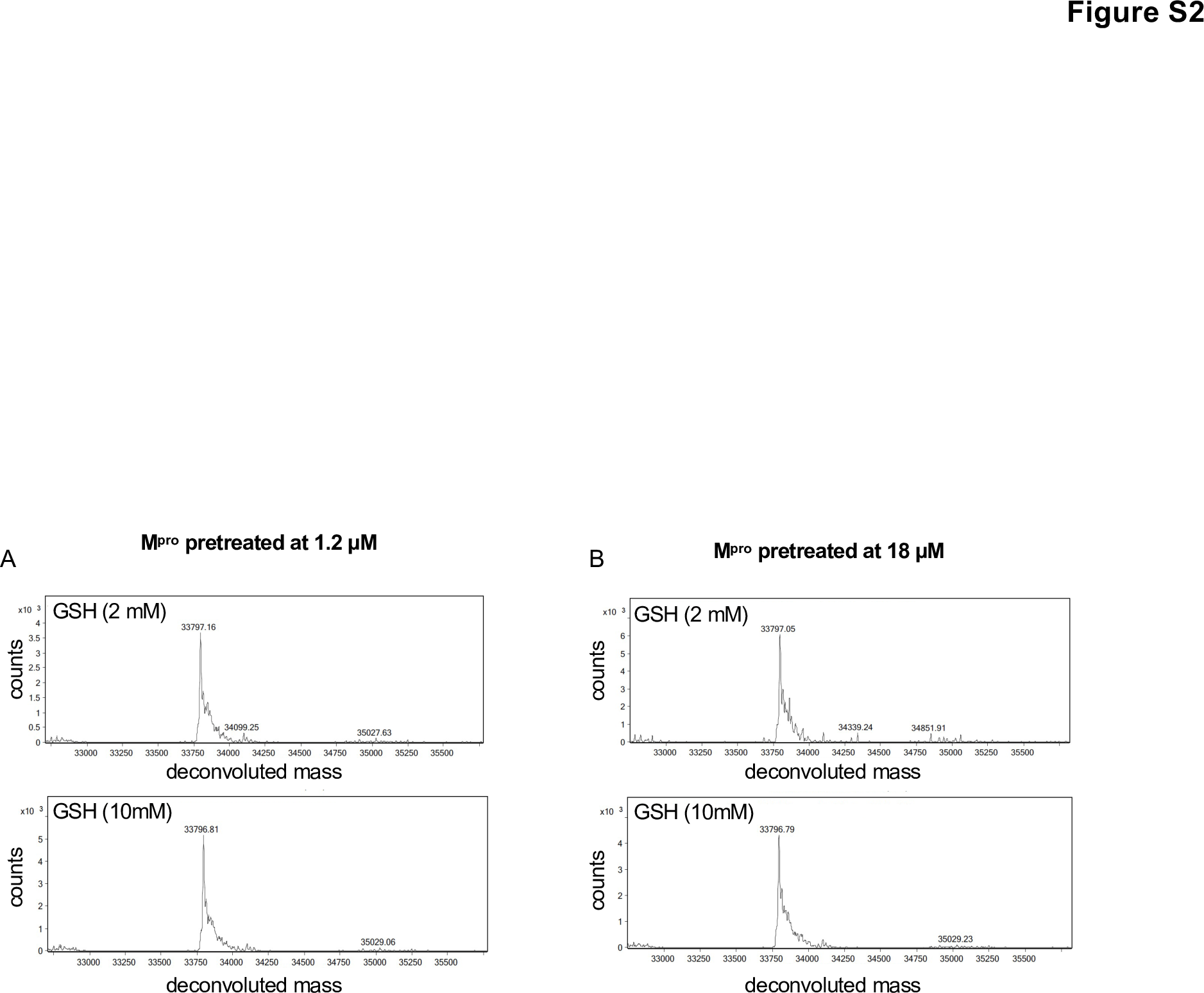

Supplement: FIG S2 [file mbio.02094-21-sf002.tif]

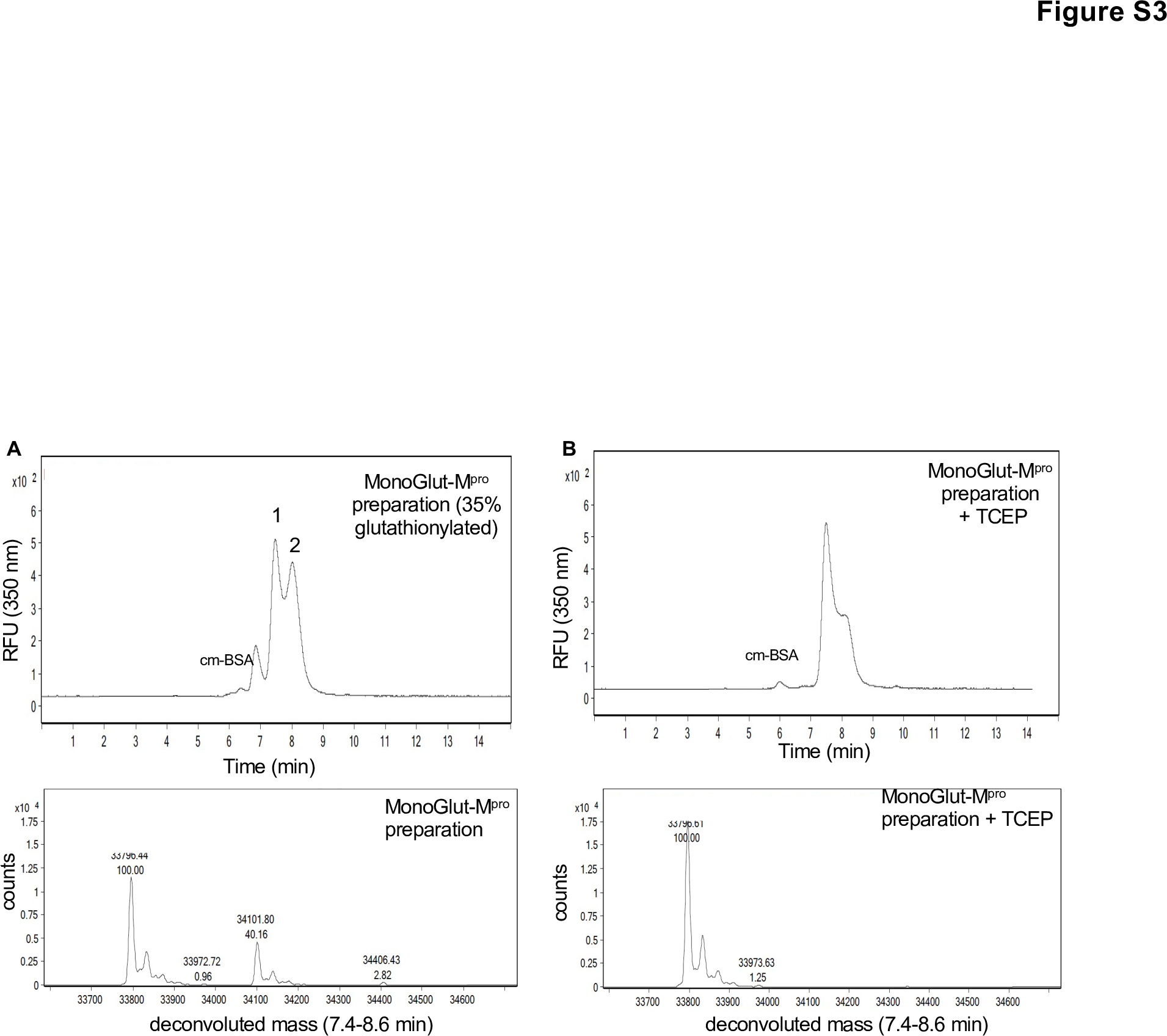

Supplement: FIG S3 [file mbio.02094-21-sf003.tif]

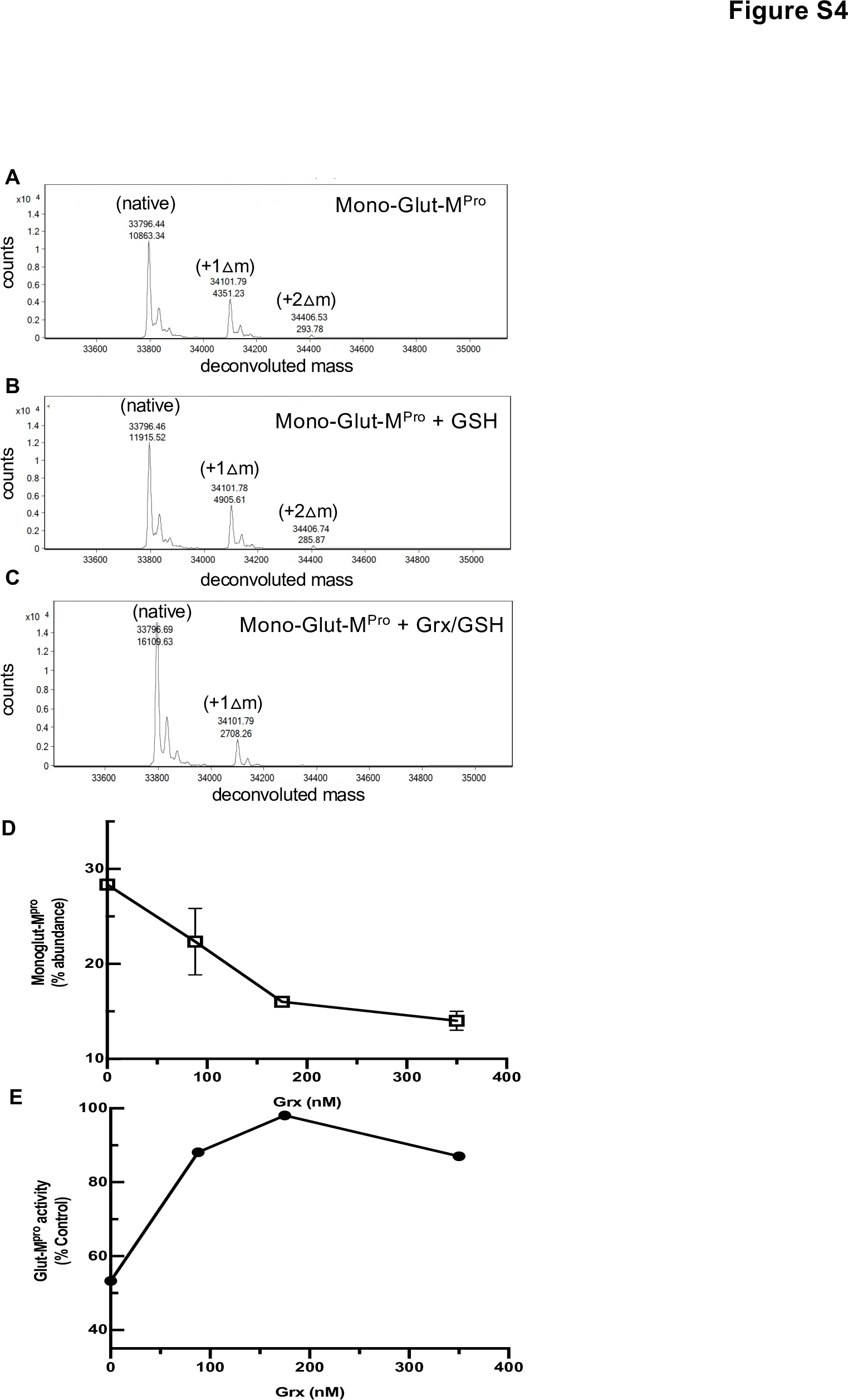

Supplement: FIG S4 [file mbio.02094-21-sf004.tif]

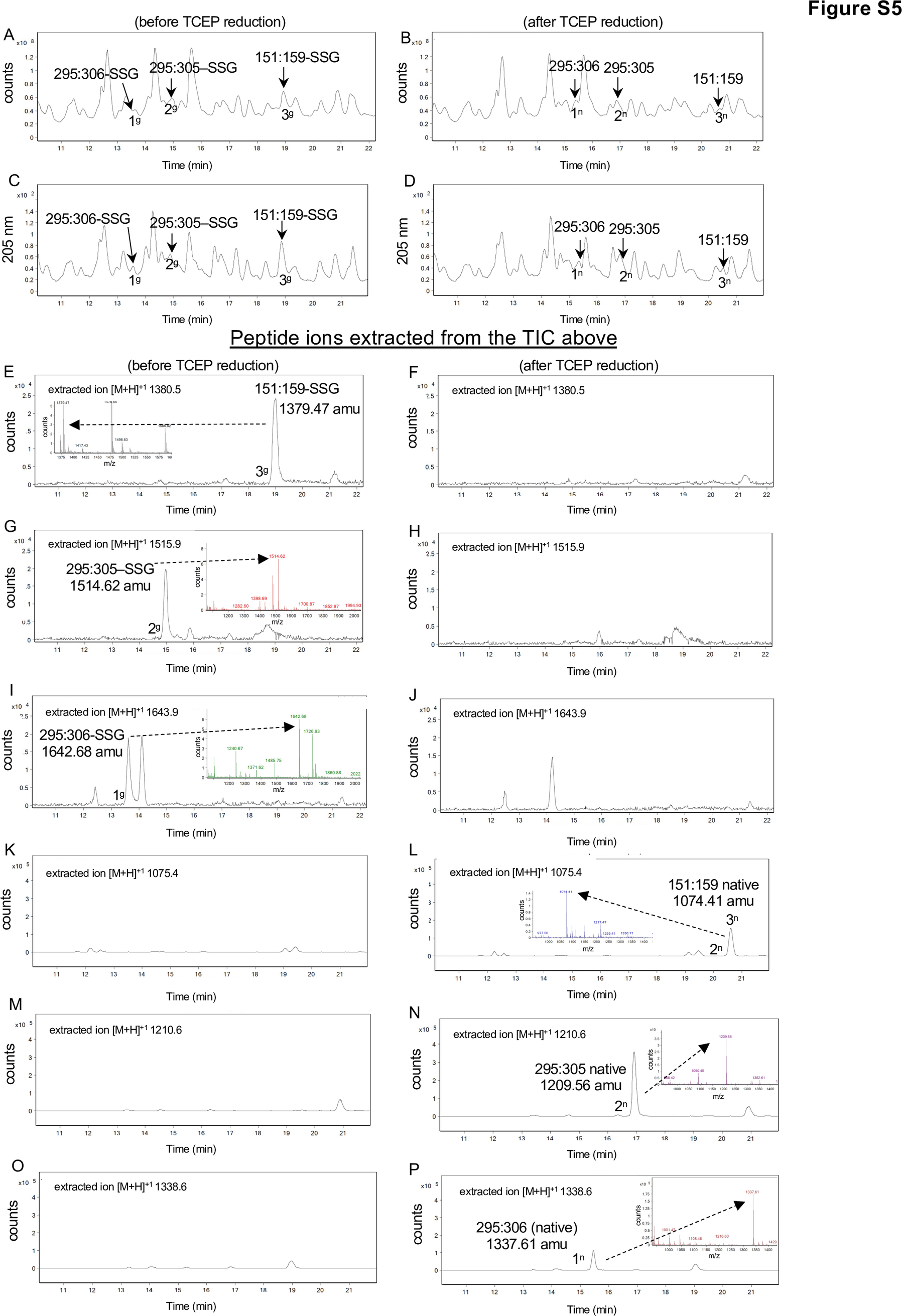

Supplement: FIG S5 [file mbio.02094-21-sf005.tif]

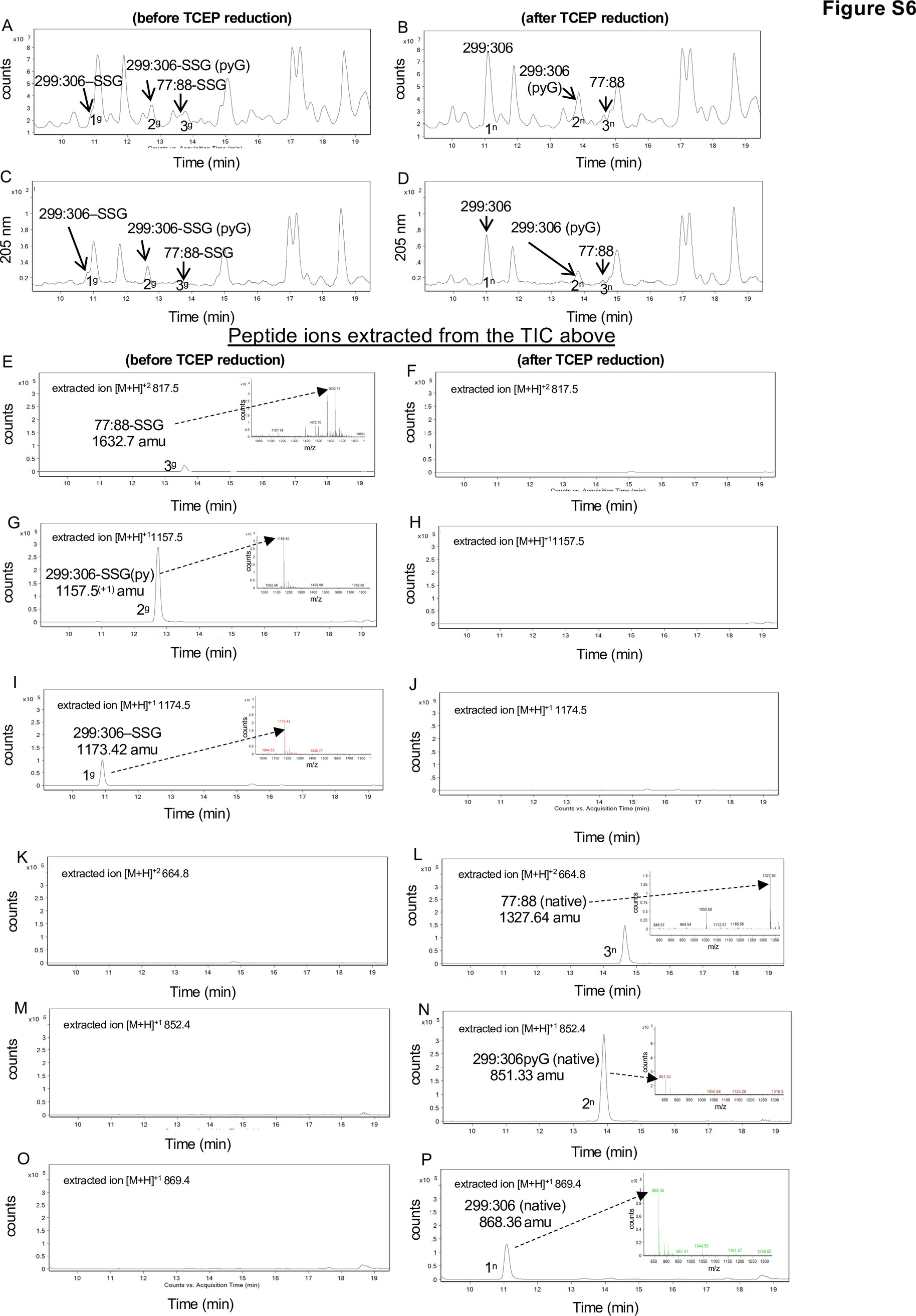

Supplement: FIG S6 [file mbio.02094-21-sf006.tif]

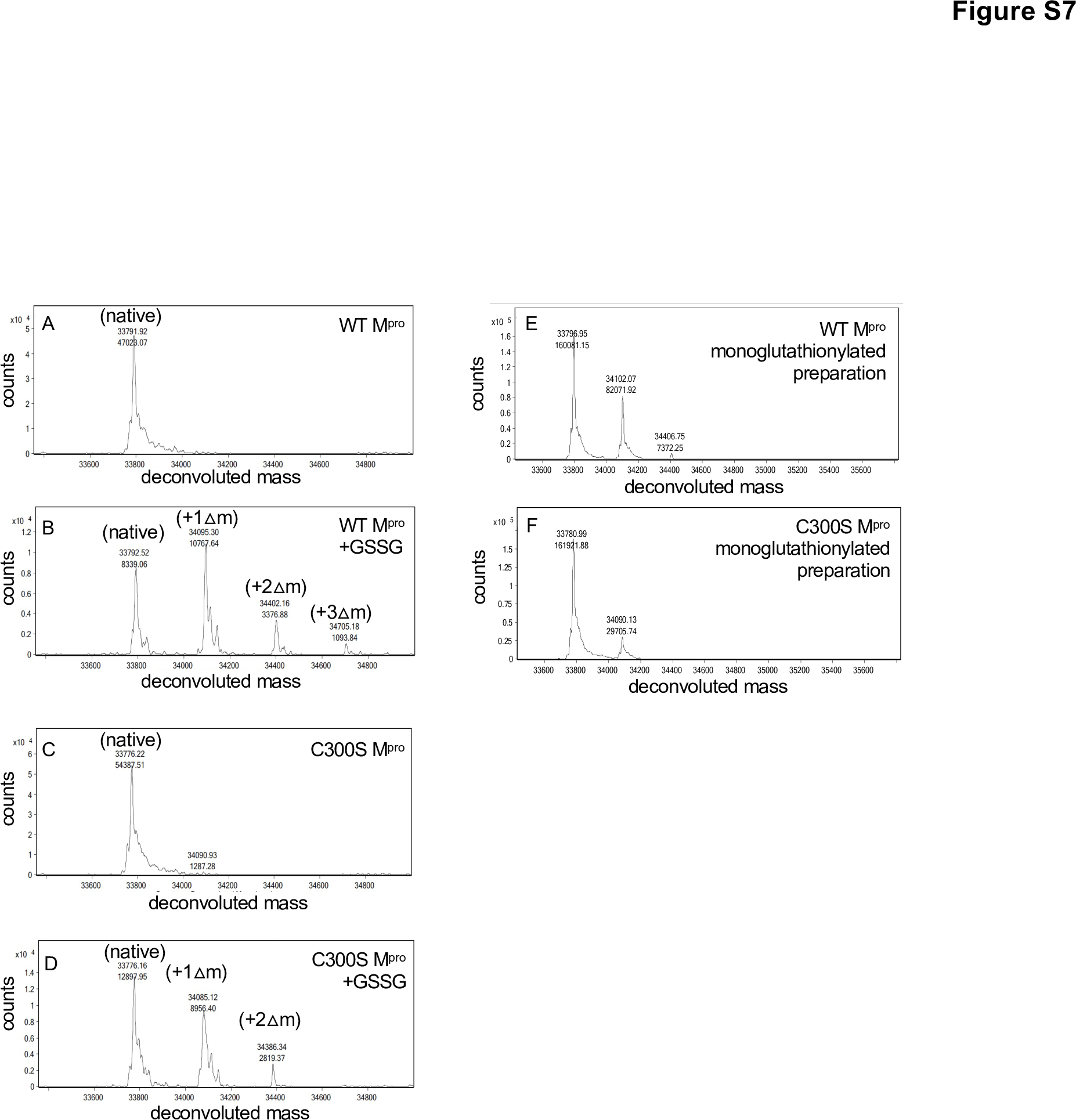

Supplement: FIG S7 [file mbio.02094-21-sf007.tif]

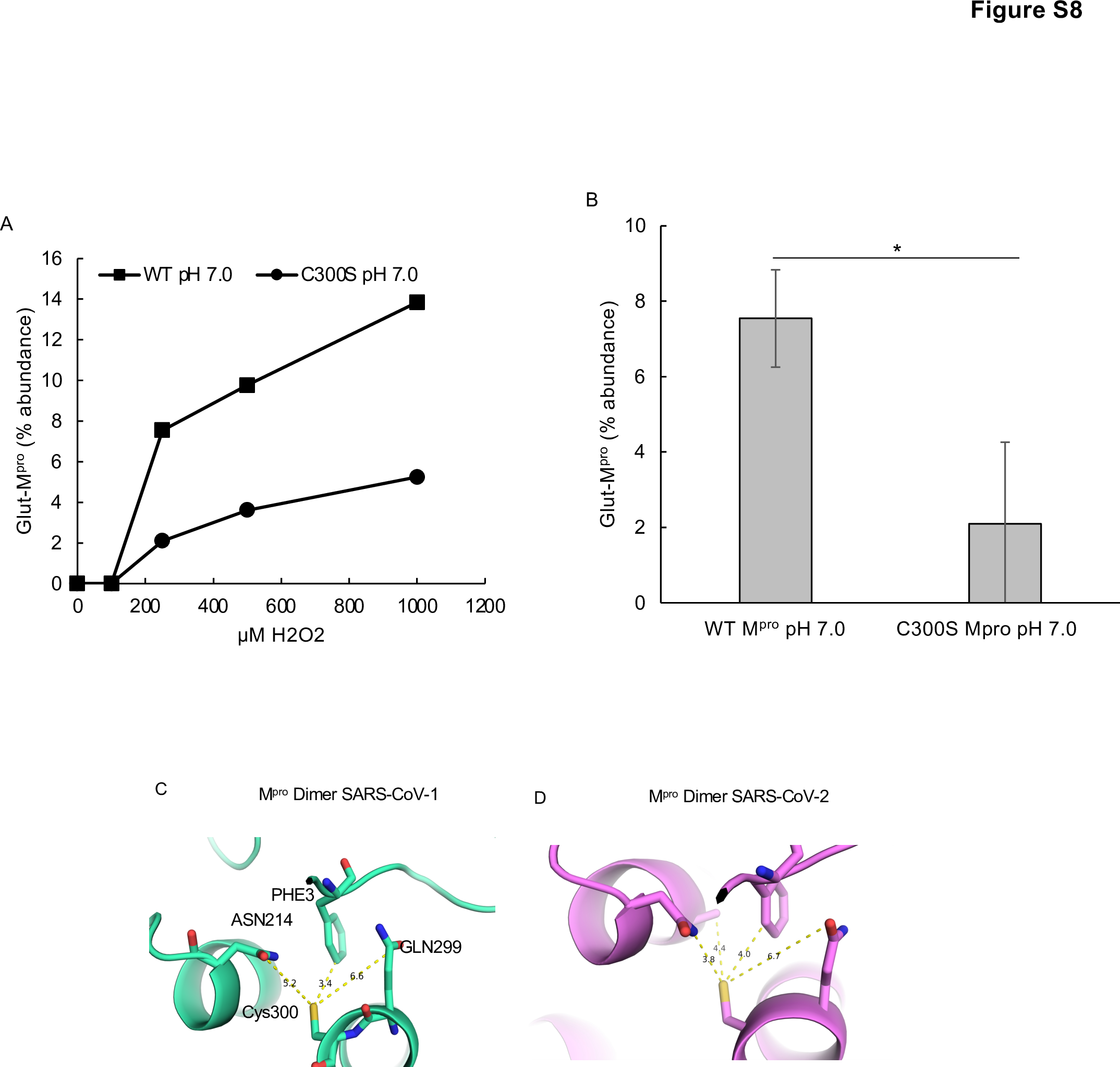

Supplement: FIG S8 [file mbio.02094-21-sf008.tif]
