# Supplementary material for: Regulation of the Dimerization and Activity of SARS-CoV-2 Main Protease through Reversible Glutathionylation of Cysteine 300
Source: mBio. 2021 Aug 17;12(4):e02094-21. doi: 10.1128/mBio.02094-21 (PMC8406260; doi:10.1128/mBio.02094-21)
Supplement: TABLE S1 [file mbio.02094-21-st001.pdf]

Table S1: RP/HPLC/MALDI-TOF MS Identification of peptides after cysteine alkylation and chymotrypsin digestion of M^{pro} or monoglutathionylated M^{pro} preparations

Peptide Number	Peptide From:To	$M_{r\ (calc)}$	$M_{r\ (expt)}$	Delta	R.T.
1	9:31 (cys16 & 22)	2630.22	ND	-	-
2	38:54 (cys38 & 44)	2240.96	2239.41.	1.55	24
3	67:101 (cys85)	3987.16	ND	-	-
41	113:118 (cys117)	779.35	779.33	-0.02	17.7
5a[1]	127:134 (cys128)	1090.47	1090.45	-0.02	16.9/17.3
5b[1]	py127:134 (cys128)	1073.47	1073.44	-0.03	18.9/19.3
61	141:150 (cys145)	1064.46	1064.44	-0.02	18.1
7a[1]	155:159 (cys156)	694.26	694.36	0.1	9.7
7b[1]	151:159 (cys156)*	1199.43	1199.46	0.03	22.5
8	160:161 (cys161)	409.13	ND	-	-
9	240:291 (cys265)	5560.84	ND	-	-
10a[1]	295:305 (cys300)	1334.63	1334.61	-0.02	19
10b[1]	295:306 (cys300)**	1462.69	1462.66	-0.03	17.4
111	141:150 (cys-sg 145)	1244.48	1240.67	-3.81	13.7
11n[1]	141:150 (native)	939.4	939.4	0	15.1
121	151:159 (cys-sg 156) *	1379.3	1379.47	0.17	19
12n[1]	151:159 (native)	1074.42	1074.41	-0.01	20.7
131	295:306 (cys-sg 300)	1642.7	1642.68	-0.02	13.7
13n[1]	295:306 (native) **	1337.63	1337.61	-0.02	15.5
141	295:305 (cys-sg 300)	1514.66	1514.62	-0.04	14.9
14n[1]	295:305 (native)	1209.58	1209.56	-0.02	16.9
15	4:8	651.34	651.35	0.01	9.7***
16	32:37	722.34	722.34	-0.02	13.3
17	55:66	1484.76	1482.75	-2.01	19.8
18	104:112	1044.56	1044.56	0	14.4
19	119:126	779.33	779.34	0.01	17.7
20	135:140	651.35	651.35	0	9.7***
21	151:154	523.22	523.23	0.01	4.1
22	162:181	2191.97	2192.95	0.02	20.8
23	186:207	2330.18	2328.94	-2.18	26
24	210:218	1000.5	1000.49	-0.01	14.7
25	220:223	548.29	548.3	0.01	8.2
26	224:230	810.37	810.36	-0.01	14.1
27	231:237	837.43	ND	-	-

Peptides 1-10 are the cysteine containing peptides predicted and, where indicated, identified after chymotrypsin digestion. Peptides 11-14 are the glutathionylated and native forms of peptides identified. Peptides 16-27 are the non-cysteine containing peptides predicted and, where indicated, identified after chymotrypsin digestion. The -sg indicates glutathionylated peptide. **These peptides containing cysteine 156 occur due to lack of cleavage at the 154:155 predicted chymotryptic cleavage site. *** These peptides containing Cys300 occur due to incomplete cleavage at the 305:306 predicted chymotryptic cleavage site. ND=Not Detected
