# Supplementary material for: Regulation of the Dimerization and Activity of SARS-CoV-2 Main Protease through Reversible Glutathionylation of Cysteine 300
Source: mBio. 2021 Aug 17;12(4):e02094-21. doi: 10.1128/mBio.02094-21 (PMC8406260; doi:10.1128/mBio.02094-21)
Supplement: TABLE S2 [file mbio.02094-21-st002.pdf]

Table S2: RP/HPLC/MALDI-TOF MS Identification of peptides after cysteine alkylation and trypsin/lysC digestion of M^{pro} or monoglutathionylated M^{pro} preparation

Peptide Number	Peptide From:To	$M_{r\ (calc)}$	$M_{r\ (expt)}$	Delta	R.T.
1	13:40 (cys16,22,38)	3446.56	3448.51	1.95	28.2
2a	41:60 (cys44)	2499.17	2500.14	0.97	24.6
2b	41:61 (cys44) *	2627.26	2628.23	0.97	23.8
3	77:88 (cys85)	1452.71	1452.7	-0.01	17.3
4a	106:131 (cys117,128)	3026.37	ND	ND	ND
4b	106:137 (cys117,128) **	3726.76	3728.7	1.94	26
5	138:188 (cys145,156, 161)	5561.38	ND	ND	ND
6	237:269 (cys265)	3699.81	3701.75	1.94	29.2
7a	299:306 (cys300)	993.41	993.41	0	15.1
7b	299:306 (cys300)(pGlu) ***	976.41	976.38	-0.03	17.2
8	77:88 (cys-sg 85)	1632.74	1632.71	-0.03	13.5
8n	77:88 (native cys 85)	1327.66	1327.64	-0.02	14.7
9	299:306 (cys-sg 300)	1173.44	1173.42	-0.02	10.9
9n	299:306 (native cys 300)	868.36	868.36	0	11.2
10	299:306 (pGlu) (cys-sg 300)	1156.44	1156.4	-0.04	12.7
10n	299:306 (pGlu) (native cys 300)	851.36	851.33	-0.03	14
11	1:4	465.22	465.23	0.01	2.9
12	6:12	736.34	736.35	-0.01	10.8
13	62:76	1695.87	1695.86	-0.01	18.7
14	91:97	734.37	ND	ND	ND
15	132:137	718.39	718.37	-0.02	8.8
16	189:217	3032.55	3032.54	-0.01	25.6
17	218:222	734.38	ND	ND	ND
18	223:236	1613.8	1613.78	-0.02	23.7
19	270:279	1130.54	1130.53	0.01	11.9
20	280:298:	2121.09	2122.07	-0.02	27

Peptides 1-7 are the cysteine containing peptides predicted and, where indicated, identified after trypsin/lysC digestion. Peptides 8-10 are the glutathionylated and native forms of peptides identified. Peptides 11-20 are the non-cysteine containing peptides predicted and, where indicated, identified after trypsin/lysC digestion. The -sg indicates glutathonylated peptide. **These peptides are the result of the spontaneous deamidation that occurs with peptides containing an N-terminal glutamine and the retention times and molecular masses for this peptide were confirmed with the use of synthetic peptides that were run on RP-HPLC/MS. The retention times (RT) and molecular masses for the Cys300 peptides were confirmed with the use of synthetic peptides that were run on RP-HPLC/MALDI-TOF as native, alkylated or glutathionylated peptides. Peptide samples were analyzed without (-) and with (+) TCEP to remove glutathione moieties. Shown are the calculated native masses [$M_{r(calc)}$] and the experimental masses [$M_{r(expt0)}$]. ND=Not Detected
